# Carbonization of MOF-5/Polyaniline Composites to N,O-Doped Carbon/ZnO/ZnS and N,O-Doped Carbon/ZnO Composites with High Specific Capacitance, Specific Surface Area and Electrical Conductivity

**DOI:** 10.3390/ma16031018

**Published:** 2023-01-22

**Authors:** Marjetka Savić, Aleksandra Janošević Ležaić, Nemanja Gavrilov, Igor Pašti, Bojana Nedić Vasiljević, Jugoslav Krstić, Gordana Ćirić-Marjanović

**Affiliations:** 1 Vinča Institute of Nuclear Science, University of Belgrade, National Institute of the Republic of Serbia, P.O. Box 522, 11001 Belgrade, Serbia; 2 Faculty of Pharmacy, University of Belgrade, Vojvode Stepe 450, P.O. Box 146, 11221 Belgrade, Serbia; 3 Faculty of Physical Chemistry, University of Belgrade, Studentski trg 12-16, 11158 Belgrade, Serbia; 4 Department of Catalysis and Chemical Engineering, Institute of Chemistry, Technology and Metallurgy, University of Belgrade, Njegoševa 12, 11000 Belgrade, Serbia

**Keywords:** N,O-doped carbon, capacitance, carbonization, composite, MOF-5, polyaniline, ZnO, ZnS, surface area

## Abstract

Composites of carbons with metal oxides and metal sulfides have attracted a lot of interest as materials for energy conversion and storage applications. Herein, we report on novel N,O-doped carbon/ZnO/ZnS and N,O-doped carbon/ZnO composites (generally named C-(MOF-5/PANI)), synthesized by the carbonization of metal–organic framework MOF-5/polyaniline (PANI) composites. The produced C-(MOF-5/PANI)s are comprehensively characterized in terms of composition, molecular and crystalline structure, morphology, electrical conductivity, surface area, and electrochemical behavior. The composition and properties of C-(MOF-5/PANI) composites are dictated by the composition of MOF-5/PANI precursors and the form of PANI (conducting emeraldine salt (ES) or nonconducting emeraldine base). The ZnS phase is formed only with the PANI-ES form due to S-containing counter-ions. XRPD revealed that ZnO and ZnS existed as pure wurtzite crystalline phases. PANI and MOF-5 acted synergistically to produce C-(MOF-5/PANI)s with high S_BET_ (up to 609 m^2^ g^−1^), electrical conductivity (up to 0.24 S cm^−1^), and specific capacitance, C_spec,_ (up to 238.2 F g^−1^ at 10 mV s^−1^). Values of C_spec_ commensurated with N content in C-(MOF-5/PANI) composites (1–10 wt.%) and overcame C_spec_ of carbonized individual components PANI and MOF-5. By acid etching treatment of C-(MOF-5/PANI), S_BET_ and C_spec_ increased to 1148 m^2^ g^−1^ and 341 F g^−1^, respectively. The developed composites represent promising electrode materials for supercapacitors.

## 1. Introduction

Increasing energy demands of modern society stimulated the investment of great efforts in developing efficient, inexpensive, sustainable, and eco-friendly electrochemical devices for energy conversion and storage (ECS), such as fuel cells, batteries, and electrochemical capacitors (supercapacitors). The development of such systems requires progress in the synthesis of advanced materials acting as their active electrode components. Research on advanced electrode materials in ECS has been devoted to various types of materials, such as heteroatom (N, B, P, O, S)-doped carbon materials, metal oxides, conducting polymers, metal–organic frameworks (MOFs), and composites containing them. Among heteroatom-doped carbons, N-, O-, and N,O-doped carbons (activated carbons, carbon blacks, graphites, pyrolytic carbons, carbon fibers, carbon nanotubes (CNTs) and other carbon nanostructures, and others) have attracted increasing attention during the past two decades [1,2,3,4]. It was found that the functionalization of carbon materials by N- and O-containing functional groups change their electronic structure and physico-chemical properties, thus improving their performance in ECS applications [1,3,5]. For example, it has been realized that the specific capacitance of N,O-doped CNTs was improved compared with that of pristine CNTs, which was attributed to introduced fast redox reactions involving N- and O-containing functionalities (i.e., to pseudocapacitive effect) [6]. Improved hydrophilicity due to the introduction of N functional groups has also been related to better capacitive behavior of N-doped CNTs [7]. It was reported that K-ion hybrid capacitors with N,O-doped hard carbons as electrode materials exhibited high reversible capacity and superior cycling stability, attributed to the coordination effect of N and O dopants providing enhanced electronic conductivity, enlarged layer spacing, and abundant active sites for K-ion storage [8,9]. N-, O-, and N,O-doped carbons were also shown to be highly active electrocatalysts for key electrochemical reactions in energy technologies, such as hydrogen evolution [10], oxygen reduction [2], and oxygen evolution reaction [11,12]. Improved electrocatalytic activity of carbon materials was attributed to N- and O-containing functional groups, which modify surface properties (reactivity and wettability) and serve as surface-active sites. Using N,O-doped carbons as electrode materials was also shown to improve the performance of various types of rechargeable batteries [13] and water-splitting devices [14]. Carbonization, frequently followed by activation, was commonly used as a simple and low-cost method to produce N-, O-, and other heteroatom-doped carbons with high specific surface area, good electrical conductivity, suitable content of heteroatoms, and improved electrochemical and other properties. Various compounds were explored as precursors for carbonization: polymers, organic compounds, metal carbides, metal–organic frameworks (MOFs), etc. [1,5,15,16].

Due to their high electrical conductivity, redox activity, relatively simple syntheses, existence in various redox and acid–base states, and other desirable properties, conducting polymers attracted great attention as materials for applications in ECS [17]. However, their pristine ordinary forms show some weaknesses, such as low specific surface area or low cyclic stability in supercapacitors. Preparations of their composites, carbonaceous derivatives, and nanostructures represent effective strategies to improve performance in various applications. During the last decade, preparation of N- and O-containing carbonaceous materials by the carbonization of conducting polymers as precursors attracted significant scientific interest, where PANI and polypyrrole (PPy), which contain N atoms, were the most frequently used [3,17,18]. N,O-containing carbonaceous materials produced from PANI and PPy precursors exhibited good electrical conductivity, pretty large specific surface area, suitable content of N and O heteroatoms and functional groups containing them, suitable pore distribution, and other properties desirable for applications in ECS [15,19,20,21,22,23,24,25,26,27]. It was found that the properties of N,O-doped carbon materials derived from conducting polymers can be tuned rather effectively by tailoring the molecular structure and morphology of the conducting polymer precursor. The gravimetric capacitance, specific surface area S_BET,_ and electrical conductivity of PANI-derived nanostructured carbons reached values up to c.a. 400 F g^−1^, 440 m^2^ g^−1^, and 0.8 S cm^−1^, respectively [27]. To improve the specific surface area, porosity, and other properties of conducting polymer-derived carbon materials, various templating techniques were applied (e.g., the addition of the hard template in the precursor for carbonization), usually followed by the postcarbonization removal of the template [19].

Another interesting class of materials relevant to this work are metal–organic frameworks (MOFs), also known as porous coordination polymers [28,29]. MOFs are formed by strong bonds between metal ions (Zn, Co, Ni, and others) and different organic linkers (based on carboxylates, imidazolates, phosphonates, etc.) [30,31]. They generally possess high surface area, large porosity, and crystallinity, which enable them to serve as precursors and simultaneously as self-sacrificing (self-degrading) hard templates for the production of (nano)porous carbon materials by the carbonization methods [32]. Different carbons materials were prepared from MOFs and showed high potential for a wide range of applications, including ECS [33]. In the process of MOF carbonization, the organic linker is a carbon source, while the metal component enables the production of metal oxides or metals. Thus, the final product of MOF carbonization in an inert atmosphere can be solely a carbonaceous material or a composite of carbonaceous material with metal oxide and/or metal, depending on the applied carbonization conditions [32,33]. Postcarbonization acid etching treatment was commonly used to remove remaining metal oxide and metal species from the carbonization product to produce solely carbon material, and this treatment also might induce an additional pore generation [33].

The carbon/metal oxide composites belong to the materials of particular interest for ECS applications [34]. Such composites combine advantages and mitigate weaknesses of both components. The low electrical conductivity of metal oxide is compensated by the high conductivity of carbon. In supercapacitors, metal oxide commonly provides the source of high specific capacitance and high energy density, while the carbon component improves rate capability and power density at high scan rates. Due to the synergistic effect, the electrochemical performance of the composite is often much better than that of its individual components [34]. MOFs were shown to be suitable precursors for the simple syntheses of carbon/metal oxide composites by carbonization, which enables the control of particle size and shape in the synthesized composite [32,35].

One of the most widely studied MOFs, which has attracted great attention due to its high surface area, large pore volume, crystallinity, and extensive applicability (e.g., in catalysis, gas storage and sensing), is Zn-containing MOF-5 [36,37,38]. It is a microporous crystalline material consisting of Zn_4_O units, which are connected by 1,4-benzenedicarboxylate (BDC) linkers forming a cubic metal–organic network Zn_4_O(BDC)_3_ [36]. Its synthesis can be conducted by a relatively simple procedure at room temperature and without the addition of base or other additives, which was first developed by Yaghi et al. [39] and improved recently regarding the optimal amount of water by Savić Biserčić et al. [38]. The carbonization of MOF-5 has been reported as a suitable route to prepare porous carbon materials [40]. It was found that the heating of MOF-5 under N_2_ or Ar atmosphere, with the maintenance of temperature at 900 °C for several hours, led to the complete transformation of MOF-5 to porous amorphous carbon [40,41,42]. By heating at a temperature higher than 600 °C, MOF-5 was first decomposed to ZnO, along with the formation of the carbonaceous phase. When the temperature was further increased to 900 °C, the vaporization of metal Zn (produced by the reduction in ZnO with carbonaceous matter) occurred so that only the carbonaceous phase remained after prolonged heating at 900 °C [40,42].

Recently, we synthesized for the first time a series of composites of PANI and MOF-5 (MOF-5/PANI) and demonstrated that some of them, synthesized under optimal conditions with 25% and 50% MOF-5 and PANI in ES form, joined key attributes of the individual components—high S_BET_ of 225 and 730 m^2^ g^−1^, respectively, originating from MOF-5, and good electrical conductivity, 1.0 × 10^−3^ S cm^−1^ and 1.4 × 10^−4^ S cm^−1^, respectively, coming from PANI. In this way, the weaknesses of each of the components—low specific surface area of PANI and low electrical conductivity of MOF-5—were overcome [43].

Continuing in this direction, with the aim of synthesizing materials with improved properties for ECS applications, herein, we developed a one-pot synthesis of novel N,O-doped carbon/ZnO and N,O-doped carbon/ZnO/ZnS composites (C-(MOF-5/PANI) by direct carbonization of PANI/MOF-5 composites. We devoted special attention to the influence of PANI/MOF-5 precursor composition (the content of MOF-5 and PANI), the type of PANI (conducting ES or nonconducting EB form) used for the preparation of the precursors, and the acid etching postcarbonization treatment on the structure and properties of the final materials. For the appropriate precursor composition, C-(MOF-5/PANI) composites and acid etching derivatives exhibited high specific capacitance, specific surface area, and electrical conductivity. To the best of our knowledge, this is the first report on the preparation of N,O-doped carbon/metal oxide and N,O-doped carbon/metal oxide/metal sulfide hybrid materials by direct carbonization of MOF/conducting polymer composites. The applied synthetic strategy could be applied in the future to a variety of MOF-conducting polymer combinations to produce advanced novel composites of heteroatom-doped carbons with metal oxide and/or metal sulfides. To demonstrate the potential applicability of these advanced materials, here, we also provide preliminary insights into their potential use as electrode materials for electrochemical capacitors.

## 2. Materials and Methods

### 2.1. Chemicals

Aniline (p.a., >99.5%, Centrohem, Stara Pazova, Serbia) was distilled under reduced pressure and stored under argon before use. Benzene-1,4-dicarboxylic acid (H_2_BDC, terephthalic acid, p.a., >98%), zinc acetate dihydrate (p.a., >99%), dimethylformamide (DMF, p.a., >99.5%), chloroform (CHCl_3_, p.a., >99%), HCl (p.a., >36.5%), ammonium peroxydisulfate (APS, analytical grade), ethanol (analytical grade), and ammonium hydroxide (analytical grade) were used as received from Centrohem, Serbia.

### 2.2. Syntheses of MOF-5/PANI Composite Precursors

Composites of MOF-5 and PANI, MOF-5/PANI, which were used as precursors to synthesize C-(MOF-5/PANI) composites, were prepared by the procedures reported in ref. [43]. Two series of precursor composites, MOF/ES and MOF/EB, were synthesized using PANI in its conducting emeraldine salt (ES) form, PANI-ES, and in its deprotonated, nonconducting emeraldine base (EB) form, PANI-EB, respectively.

Zn-containing MOF-5, used for the preparation of MOF-5/PANI precursor composites, was synthesized by the basic procedure developed by Yaghi’s group [39], modified by our group, Savić Biserčić et al. [38], so that anhydrous zinc acetate (Zn(OAc)_2_) was used as the starting precursor instead of zinc acetate dihydrate, and the optimal quantity of water (mole ratio H_2_O/Zn^2+^ = 0.5) was added. Thus, Zn(OAc)_2_ × 0.5H_2_O was in situ prepared, enabling the synthesis of completely pure crystalline, white powder MOF-5. The prepared solution of Zn(OAc)_2_ × 0.5H_2_O was poured into a solution of terephthalic acid in DMF at room temperature, and the reaction mixture was stirred for 1 h, after which the precipitate was filtered, transferred into DMF and left overnight. Final activation was performed by solvent exchange with chloroform, followed by heating of produced MOF-5 in a vacuum, as described in ref. [38].

The precursor composites of MOF/ES series were prepared by the mechano-chemical route, according to the procedure reported in ref. [43]. PANI-ES component of MOF/ES composites was synthesized by the oxidation of aniline hydrochloride (0.2 M) with the oxidant APS (0.25 M) in an aqueous medium. The solution of APS was poured into the solution of aniline hydrochloride, and the reaction mixture was stirred for about 3 min, and then it was left for 50 min at room temperature. The precipitated PANI-ES was collected by filtration, washed with distilled water and ethanol, and first dried in air at room temperature and then in a vacuum at 60 °C for 3 h. Three MOF/ES composites, MOF/ES-1, MOF/ES-2, and MOF/ES-3, were synthesized using initial mass percents of 25%, 50%, and 75% of MOF-5, respectively [43]. Measured amounts of MOF-5 and PANI-ES were placed in a mortar with a small volume of chloroform, crushed, and mixed with a pestle until total evaporation of the solvent. Then, the obtained MOF/ES was dried in a vacuum, first at room temperature and then at 120 °C, purged with argon and stored in a desiccator. The real content of MOF-5, determined by measuring Zn concentration in composites using the FAAS technique, was very close to that theoretically predicted, amounting to 25.2, 50.7 and 77.2 wt.% for MOF/ES-1, MOF/ES-2, and MOF/ES-3, respectively [43].

The precursor composites of MOF/EB series were prepared by mixing the dissolved part of PANI-EB in DMF with MOF-5 in different mass ratios, according to the procedure described in ref. [43]. For that purpose, PANI was first synthesized in ES form by the oxidative polymerization of aniline (0.2 M) with APS (0.25 M) in water without added acid, and then it was deprotonated by an excess of 5% NH_4_OH to produce PANI-EB. Three composites of this series, denoted as MOF/EB-1, MOF/EB-2, and MOF/EB-3, were prepared by pouring dispersion of a specified amount of MOF-5 in DMF into the solution containing the appropriate amount of dissolved PANI-EB in DMF to achieve mass percentages of 25%, 50%, and 75% of MOF-5, respectively, and stirring the obtained mixture for 3 h [43]. After filtering, the obtained composite precipitate was immersed in chloroform, and this dispersion was left overnight. Finally, the precipitate was collected by filtration, washed with chloroform, and dried in a vacuum, first at room temperature and then at 120 °C. The real content of MOF-5 in composites, determined by measuring Zn concentration in composites by FAAS, amounted to 71.0, 77.4, and 89.0 wt.% for samples MOF/EB-1, MOF/EB-2, and MOF/EB-3, respectively [43].

### 2.3. Syntheses of C-(MOF-5/PANI) Composites, C-MOF, C-PANI-ES, and C-PANI-EB

Direct carbonization of MOF-5/PANI composite precursors was performed at a heating rate 10 °C/min up to the temperature of 800 °C in an argon environment at a flow rate of 120 cm^3^/min, using a horizontal tube furnace (Elektron, Beograd, Serbia) to produce C-(MOF-5/PANI) composites. The composites produced by carbonization of MOF/ES-1, MOF/ES-2, and MOF/ES-3 precursors are denoted as C-MOF/ES-1, C-MOF/ES-2, and C-MOF/ES-3, respectively, while the composites produced by carbonization of MOF/EB-1, MOF/EB-2, and MOF/EB-3 precursors are denoted as C-MOF/EB-1, C-MOF/EB-2, and C-MOF/EB-3, respectively.

For comparison, individual components of MOF-5/PANI precursors, MOF-5, PANI-ES, and PANI-EB, were carbonized under the same conditions as described for composites, and their products of carbonization are denoted as C-MOF, C-PANI-ES, and C-PANI-EB, respectively.

### 2.4. Postsynthetic Acid Etching Treatment

To reduce the content of the Zn-containing phase and evaluate possible effects on the material’s properties, the selected as-prepared C-(MOF-5/PANI) samples and C-MOF were subjected to postcarbonization acid etching treatment with concentrated H_2_SO_4_. A portion of the powder sample (150 mg) was treated with 0.5 M H_2_SO_4_ (5 mL) for 2 h at room temperature, with occasional mixing using a stirring rod. Then, the dispersion was centrifuged, and the isolated precipitate was washed several times with distilled water to achieve pH 6–7, followed by drying in air, first at room temperature for 24 h and then at 100 °C for 1 h. Codes of materials obtained after this treatment contain the suffix “a” added to the name of the corresponding as-prepared product of carbonization (e.g., C-MOF/ES-1-a).

### 2.5. Characterization of Synthesized Materials

The elemental microanalysis (C, N, H) was carried out using the Elemental Analyzer Vario EL III (Elementar, Langenselbold, Germany). The morphology of the samples was examined by a scanning electron microscope (SEM) equipped with an energy-dispersive X-ray spectroscopy (EDX) system, a PhenomProX SEM-EDX (Phenom, Rotterdam, The Netherlands). Before SEM measurements, the sample was covered with a very thin layer of copper using a Mini plasma sputtering coater (Zhengzhou CY Scientific Instruments, Zhengzhou, China). EDX was used to analyze the elemental composition of the sample surface/subsurface. For this purpose, samples were not coated with copper. Zn content in the composites was determined by flame atomic absorption spectroscopy (FAAS), using atomic absorption spectrometer Perkin-Elmer AAnalyst 700, Waltham, MA, USA. Prior to FAAS measurements, the microwave-assisted acid digestion of the samples was performed using ETHOS 1 Advanced Microwave Digestion System (Milestone, Italy). The acid mixture consisting of 65% HNO_3_ (2 mL) and 36% HCl (6 mL) was added to c.a. 15 mg of the sample in a Teflon vessel. The closed vessel was fitted on a digestion carousel and heated for 20 min at a constant temperature of 210 °C after the previous 15 min needed to reach that temperature. When cooled to room temperature, the obtained solution was diluted by deionized water to 25 mL and was ready for determination of zinc concentration by FAAS. The thermal analysis (TGA and DTA) of the samples, placed in platinum pans, was carried out using a TA Instruments Model SDT 2960 thermoanalytical device (New Castle, DE, USA) with air purging gas at a flow rate of 80 mL min^−1^ and a heating rate of 10 °C min^−1^. The X-ray diffraction (XRD) patterns of the powdered samples were recorded on a Rigaku Ultima IV diffractometer with Ni-filtered CuKα radiation (λ = 1.54178 Å, 40 kV, 40 mA). The following diffraction settings were selected: diffraction angle 2θ between 5° and 85°, step size of 0.05°, and acquisition time of 2°/min. The electrical conductivity of the powdered samples compressed between two stainless steel pistons, within an isolating hard-plastic die, was measured at room temperature using LCR meter, model LCR-6100 (GW Instek, New Taipei City, Taiwan), at room temperature and a fixed frequency of 1.0 kHz. Using a manual hydraulic press, pellets were kept under the constant pressure of 255 MPa during the measurements. FTIR spectra of the powdered samples, dispersed in KBr and compressed into pellets, were recorded in the wavenumber range of 4000–400 cm^−1^ with 64 scan per spectrum and 2 cm^−1^ resolution using the Nicolet iS20 FTIR Spectrometer (Thermo Scientific, Waltham, MA, USA). Raman spectra of the powdered samples were collected using DXR Raman microscope (Thermo Scientific, Walthman, MA, USA), equipped with a research optical microscope and a CCD detector. A diode-pumped solid-state high-brightness laser with the excitation wavelength (λ_exc_) of 532 nm and laser power on the sample of 2 mW was applied for recording all the spectra. The powdered sample was placed on an X–Y motorized sample stage. The laser beam was focused on the sample using an objective magnification of ×10. The scattered light was analyzed by the spectrograph with a 900 lines of mm^−1^ grating. The spectra were recorded using 10 s exposure time and 10 exposures per spectrum. The isotherms of the studied materials were obtained by nitrogen adsorption at 77 K using a Sorptomatic 1990 Thermo Finnigan device. Prior to adsorption, the samples were degassed for 4 h at 90 °C under vacuum and additionally 16 h at 200 °C at the same residual pressure. The Brunauer–Emmett–Teller (BET) method was used to determine specific surface area, S_BET_. The electrochemical behavior of the synthesized samples in 6M KOH aqueous solution was explored by cyclic voltammetry using Ivium VO1107 potentiostat/galvanostat (Eindhoven, Netherlands) in a standard three-electrode cell with saturated calomel electrode (SCE) and Pt foil as reference and counter electrode, respectively. Glassy carbon (GC) electrode surface, polished to a mirror finish with alumina paste, was used to support the ink prepared by dispersing 5 mg of the investigated sample (or 4 mg sample/1 mg Vulcan XC72) in ethanol/water/Nafion (200 μL/295 μL/5 μL) mixture and homogenizing it in an ultrasonic bath for 30 min. The desired suspension volume was drop-casted to attain the desired loading of 250 or 500 μg cm^−2^. Alcohol/water solvent evaporated under a gentle nitrogen stream after 10 min. A gentle nitrogen gas stream was kept just underneath the electrolyte surface to keep the solution free of oxygen. The mass-specific capacitance (*C_spec_*, in F g^−1^) was calculated using the equation:

$$C_{spec}=\frac{\int^{\;}i\cdot dt}{2\cdot m\cdot \Delta V}$$

where the integral constitutes the total charge passed, *i* is current, *t* is the time, Δ*V* is the explored potential window, and *m* is the mass of the active material resting on the GC electrode. Measurements were performed at ambient temperature.

## 3. Results

### 3.1. Carbonization Yield and the Bulk Elemental Composition of C-(MOF-5/PANI) Samples Determined by the Elemental Microanalysis and FAAS

**Figure 1:**
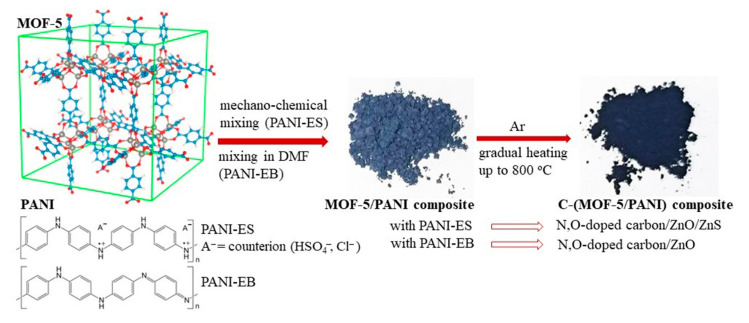
Schematic illustration of the preparation of C-(MOF-5/PANI) composites (photos refer to MOF/EB-2 precursor and C-MOF/EB-2 synthesized by its carbonization).

Carbonization of gray-colored MOF-5/PANI composites (whose color becomes lighter with increasing MOF-5 content [43]) led to black C-(MOF-5/PANI) materials (see photos in Figure 1).

The data on the yield of carbonization product, calculated as the mass of dry carbonization product C-(MOF-5/PANI) per mass of dry MOF-5/PANI precursor × 100%, are presented in Table 1. For the C-MOF/ES series, the carbonization yield was in the range 40.2–48.6% and decreased with increasing the amount of MOF-5 in the precursor. A similar range of yield was obtained for the C-MOF/EB series, 43.2–48.2%, but the yield increased with the amount of MOF-5 in the precursor.

The data on the bulk elemental composition of C-(MOF-5/PANI) samples, determined by the elemental microanalysis (C, H, and N), FAAS technique (Zn), and by difference (O), are shown in Table 1. One can see that C is the dominating element in the C-MOF/ES series, while Zn prevails in the C-MOF/EB series. The content of C decreases within the same series going from sample “1” to sample “3”, which is consistent with decreasing PANI (increasing MOF-5) content in corresponding MOF-5/PANI precursors. For the C-MOF/ES series, the content of C is significantly higher (43.4–65.61 wt.%) than for the C-MOF/EB series (28.84–35.82 wt.%). The highest content of C was found for the samples C-MOF/ES-1 (65.61 wt.%) and C-MOF/ES-2 (53.97 wt.%). The samples C-MOF/EB-2 and C-MOF/EB-3 have almost the same carbon content (c.a. 28.9 wt.%). Similarly to the trend observed for C, the content of N decreases in each series with decreasing PANI content in the MOF-5/PANI precursor (from sample “1” to sample “3”), which was to be expected since the PANI component of the composite precursors is the only source of N. The bulk N content of C-(MOF-5/PANI) samples was in the range of 0.95–9.95 wt.% and is significantly higher for the samples of the C-MOF/ES series (4.64–9.95 wt.%) compared with the samples of the C-MOF/EB series (0.95–2.43 wt.%), similarly to what was found for carbon. The samples C-MOF/ES-1 and C-MOF/ES-2 have the highest content of N among all the samples, 9.95 wt.% and 8.30 wt.%, respectively. These values are similar to those found for N,O-containing carbons obtained by the carbonization of various PANI salts (≈9–10 wt.%) [22,25,27]. Based on previous results on carbonized PANIs [27], N is present in C-(MOF-5/PANI)s as covalently bonded within the carbonaceous phase, included in various functional groups, such as pyridinic N (including phenazine type), quaternary N, pyrrolic N, N-oxide species or tetrahedral N bonded to sp^3^ C. The content of Zn was lower for the C-MOF/ES series (7.76–31.34 wt.%) than for the C-MOF/EB series (41.53–44.9 wt.%) and increased in each series going from sample “1” to sample “3”. These trends are consistent with changes in the content of MOF-5 in MOF-5/PANI precursors (see Experimental part) and are expected since MOF-5 is the only source of Zn. The samples C-MOF/EB-2 and C-MOF/EB-3 have a very similar content of Zn. Low hydrogen content (0.8–1.5 wt.%) is observed for all C-(MOF-5/PANI) samples. It is much lower than the H content of ordinary PANI-EB (~5 wt.%) and indicates a high degree of graphitization of MOF-5/PANI composites for the applied carbonization conditions. Graphitization can occur through the condensation of benzene rings (present in ordinary PANI segments and BDC linkers of MOF-5) and N- and O-containing heterocyclic rings (such as phenazine-type and phenoxazine-type units, present in the PANI component of composite precursor or formed during the carbonization) [22,23,24,25,44] accompanied with the removal of H. The content of O in C-(MOF-5/PANI) samples was in the range of 15.15–24.54 wt.%. These values are higher than those reported for carbonized PANIs [22,24,25]. This feature can be explained by the O present within the ZnO phase (as proved by XRPD, see Section 3.4), which is formed from the MOF-5 component of precursor during carbonization, apart from O, which can be incorporated covalently in graphitic structures via condensed O-containing heterocyclic rings (phenoxazine, etc.) [22] and/or functional groups such as C=O, COOH, and C-O-C [20,27].

### 3.2. The Elemental Composition of C-(MOF-5/PANI) Samples Determined by EDX

**Figure 2:**
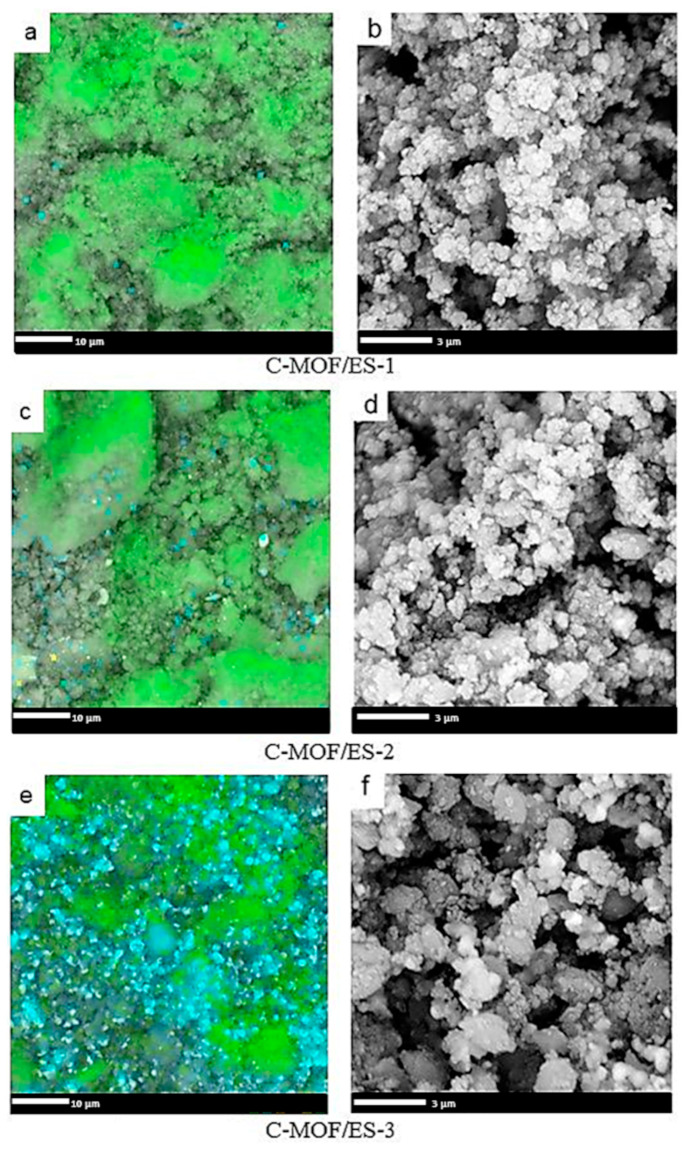
EDX elemental maps (**a**,**c**,**e**) and SEM images (**b**,**d**,**f**) of C-(MOF-5/PANI) samples of the C-MOF/ES series: (**a**,**b**) C-MOF/ES-1, (**c**,**d**) C-MOF/ES-2, and (**e**,**f**) C-MOF/ES-3. The scale bar on EDX and SEM images is 10 μm and 3 μm, respectively. The most abundant elements in EDX maps, C and Zn, are marked with green and turquoise, respectively.

**Figure 3:**
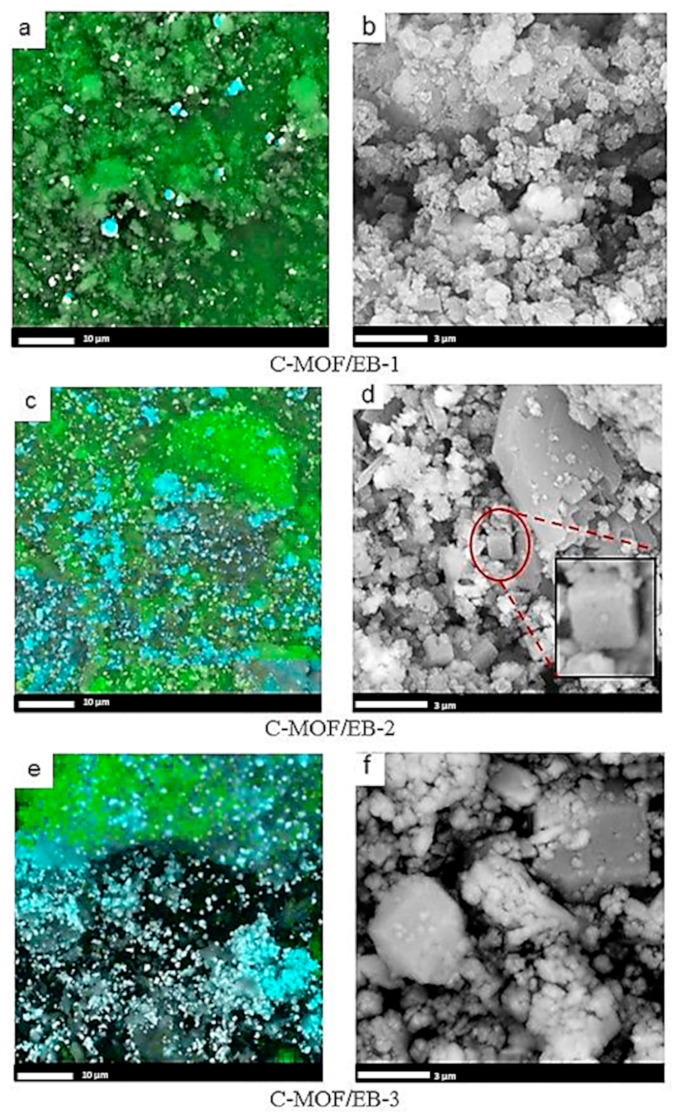
EDX elemental maps (**a**,**c**,**e**) and SEM images (**b**,**d**,**f**) of C-(MOF-5/PANI) samples of the C-MOF/EB series: (**a**,**b**) C-MOF/EB-1, (**c**,**d**) C-MOF/EB-2, and (**e**,**f**) C-MOF/EB-3. The scale bar on EDX and SEM images is 10 μm and 3 μm, respectively. The most abundant elements in EDX maps, C and Zn, are marked with green and turquoise, respectively.

The EDX spectra of C-(MOF-5/PANI) samples are shown in Figure S1, while corresponding elemental maps showing the areal distribution of elements are presented in Figure 2 (C-MOF/ES series) and Figure 3 (C-MOF/EB series). The spectra of all C-(MOF-5/PANI) samples contain peaks of C, N, Zn, O, and S. Weak peaks of Cl appear only in the spectra of C-MOF/ES series due to Cl^−^ dopant ions from HCl, which were used in the synthesis of the PANI-ES component of MOF/ES precursors. The strongest peak in all EDX spectra corresponds to C, revealing that it is the dominant element in the analyzed areas of all C-(MOF-5/PANI) materials. This finding partly differs from the results obtained by the elemental analysis (working on the principle of sample combustion and its complete conversion to gases), which showed that C is the dominant element in the C-MOF/ES series and Zn is the dominant element in C-MOF/EB series (Section 3.1). The explanation for this difference (and differences observed regarding other elements, described in further text) can be found in the fact that the microanalysis by elemental analyzer is a bulk technique that analyses the total amount of the sample (order of milligrams in our case). In contrast, in the EDX technique, the analyzed specimen is derived only from the surface and subsurface layers at the selected location of the investigated material on which the electron beam is focused. Thus, unlike the elemental analyzer, EDX measurements are dependent on the sample homogeneity and the homogeneity of the area chosen for mapping. The peaks due to Zn and O indicate the presence of ZnO [45], which is formed via the carbonization of the MOF-5 part in MOF-5/PANI composites. The data on the elemental composition of C-(MOF-5/PANI) samples obtained from EDX mapping are presented in Table 2.

The content of C determined by EDX showed values in similar ranges for both series: c.a. 57.9–64.2 wt.% for C-MOF/ES and 57.1–74.8 wt.% for C-MOF/EB samples series. For C-MOF/ES samples, surface/subsurface (EDX) and bulk contents of C have quite similar values (Table 1 and Table 2). However, for the samples of the C-MOF/EB series, C content acquired by EDX is about two times higher (57.1–74.8 wt.%, Table 2) than the corresponding bulk C content (28.8–35.8 wt.%, Table 1). As observed with the bulk C content, the content of C determined by EDX decreases with increasing the content of MOF-5 in composite precursors within both C-(MOF-5/PANI) series. This feature is seen through changes in the green-colored area representing locations of C in the elemental maps shown in Figure 2 and Figure 3. The surface/subsurface content of N also decreases with the increasing content of MOF-5 in the composite precursors. Interestingly, EDX measured significantly higher N content than the bulk N content measured by the elemental analyzer (Table 1) for all C-(MOF-5/PANI) samples. This feature could indicate that the carbonized PANI fraction in composites, which contains N covalently bonded with C in the graphitized structures, is placed (on average) to a greater extent in the surface/subsurface area than in bulk. The content of Zn determined by EDX increases within each C-(MOF-5/PANI) series with increasing the content of MOF-5 in MOF-5/PANI precursors as expected, since Zn originates from MOF-5. Except for the sample C-MOF/ES-1, for which the results from both techniques are similar, the content of Zn determined by EDX is lower (C-MOF/ES series) or significantly lower (C-MOF/EB series) than the bulk Zn content determined by FAAS. This finding indicates that the Zn-containing phases (ZnO and ZnS, as revealed by XRPD, see Section 3.4) are located to a higher extent in bulk than in the surface/subsurface areas. Unlike the elemental microanalysis, EDX measurements detected S, which is present in relatively small amounts. The S content is higher for the samples of the C-MOF/ES series (0.55–0.78 wt.%) than for C-MOF/EB series (0.12–0.43 wt.%) and decreases in each series with decreasing the amount of PANI in the precursors. The presence of S in the C-MOF/ES series is explained by the presence of the ZnS phase (which was proved by XRPD, Section 3.4) formed from S-containing counter-ions (HSO_4_^−^, SO_4_^2−^, Figure 1) in the PANI-ES component of precursors. Very small amounts of S in the C-MOF/EB series could be due to S bonded to C in the carbonaceous phase, originating from the small amount of S in o-aminoaryl sulphate or sulfonated aniline units in the PANI-EB part of MOF/EB composite precursors [46,47]. Such S-containing species cannot be removed by treating PANI-ES with the excess of alkali when it is transforming to PANI-EB, which is a characteristic feature of PANI synthesized in water without added acid [46]. Higher content of S in samples of the C-MOF/ES series is expectable, as the PANI-ES part in their precursors contains a larger amount of S present within the dopant ions. The content of O determined by EDX (Table 2) is lower for all C-(MOF-5/PANI) samples than the bulk O content determined by difference, using the elemental microanalysis and FAAS data (Table 1), similarly to what was found for Zn. By using the elemental compositions of C-(MOF-5/PANI) samples from Table 1 and Table 2 and taking into account the results of XRPD analysis, which revealed the presence of ZnO and ZnS phases in the C-MOF/ES series and ZnO phase in the C-MOF/EB series (Section 3.4), we confirmed that the part of total O is incorporated into the carbonaceous phase of C-(MOF-5/PANI) samples (besides the part of O which is present in ZnO phase). The description of this calculation, calculated O contents in carbonaceous and ZnO phases, and calculated Zn content in ZnO and ZnS phases are presented in the Supplementary Materials, Tables S1 and S2.

### 3.3. Morphology of C-(MOF-5/PANI) Composites—SEM

The morphology of C-(MOF-5/PANI) samples was explored by SEM (Figure 2 for C-MOF/ES series and Figure 3 for C-MOF/EB series). The samples of both series showed cuboid sub-micro- and microparticles and much smaller granular particles, which cover the surface of cuboid particles to a certain extent. The morphology of C-(MOF-5/PANI) samples looks quite similar to the morphology of MOF-5/PANI precursors. The cuboid shape of particles in C-(MOF-5/PANI) samples originates from the cube-shaped crystalline MOF-5 particles in the composite precursors [38,43], and accordingly, a larger amount of cuboid particles was observed in the samples synthesized from the precursors in which MOF-5 was dominating (>70 wt.%) over PANI: C-MOF/ES-3, C-MOF/EB-1, C-MOF/EB-2, and C-MOF/EB-3. On the other hand, granular morphology is dominant over cuboidal in the samples C-MOF/ES-1 and C-MOF/ES-2.

### 3.4. Crystalline Structure of C-(MOF-5/PANI) Samples—XRPD

**Figure 4:**
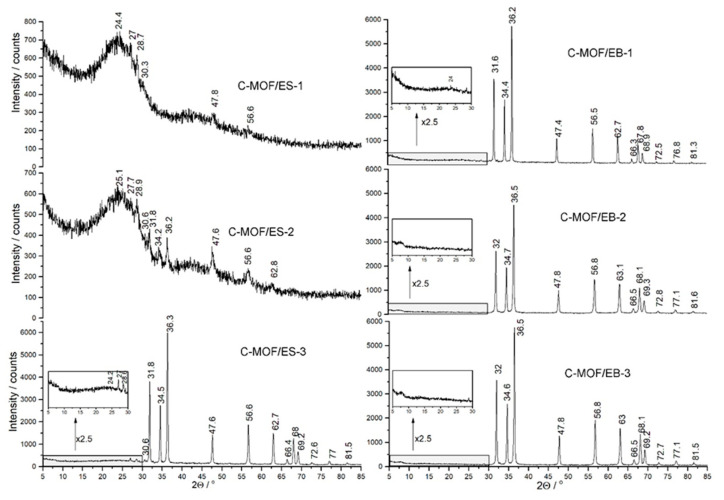
XRPD patterns of C-(MOF-5/PANI) composite samples of the C-MOF/ES series (**left**) and C-MOF/EB series (**right**).

The XRPD patterns of C-(MOF-5/PANI) composites are shown in Figure 4. The table with phase assignation and indexing of reflections is placed in the Supplementary Materials (Table S3). Characteristic reflections of crystalline ZnO are observed in the diffractograms of all composite samples except C-MOF/ES-1. The diffractograms of all three samples of the C-MOF/EB series (Figure 4 right), and the diffractogram of the sample C-MOF/ES-3 prepared from the precursor with the highest content of MOF-5 (Figure 4 left), are mutually very similar and show the sharp peaks assigned to the hexagonal wurtzite ZnO structure at 2θ of c.a. 31.8°, 34.5°, 36.3°, 47.6°, 56.6°, 62.7°, 66.4°, 68.0°, 69.2°, 77.0°, and 81.5°, originating from lattice planes (100), (002), (101), (102), (110), (103), (200), (112), (201), (202), and (104), respectively [48,49]. The lattice parameters for the hexagonal ZnO phase were determined using the formula to amount a = 3.22(2) Å and c = 5.20(2) Å. These values are in very good agreement with the literature values [48,50]. Additionally, the XRPD pattern of the sample C-MOF/ES-3 provides a few small reflections at 2θ ≈ 27°, 28.5°, and 30.6° ascribed to ZnS wurtzite-type structures (100), (002), and (101), respectively [51]. The other reflections from the ZnS phase coincide with ZnO reflections. For example, the reflection of ZnO at 2θ = 47.6 ° is also from the (110) plane in the ZnS phase, and the reflection of ZnO at 56.6° is also from the (112) plane in the ZnS phase.

$$\frac{1}{d^{2}}=\frac{4}{3}\left(\frac{h^{2}+hk+k^{2}}{a^{2}}\right)+\frac{l^{2}}{c^{2}}$$

A very broad reflection centered at 2θ ≈ 24° dominates the XRPD patterns of the C-MOF/ES-1 and C-MOF/ES-2 samples. This peak is characteristic of the amorphous carbons and corresponds to the reflection of the graphitic plane (002) [52,53]. This feature agrees well with the results from TGA that the samples C-MOF/ES-1 and C-MOF/ES-2 have the highest content of carbonaceous phase among all prepared composites (77.83 wt.% and 65.67 wt.%, respectively, see Section 3.5,). The broad peak at 2θ around 24° was previously reported as the dominant peak in the XRPD patterns of several N-containing carbon materials produced by the carbonization of PANIs, indicating domination of the disorder carbon phase [18,20,25]. The sample C-MOF/ES-2 shows reflections from ZnS and ZnO phases that emerge from the noisy pattern. The sample with the lowest content of the noncarbonaceous phase (6.97 wt.%, see Section 3.5), C-MOF/ES-1, shows small reflections attributed only to the ZnS phase, since the most intensive reflections from the ZnO phase are missing (i.e., (100), (002), and (101), which should appear in the 31°–36° 2θ region). Insets in Figure 4 are shown due to the very high intensity of ZnO reflections to enlarge the region where the main reflection of amorphous carbon is expected. There are small peaks of the amorphous carbon phase in the diffractograms of the samples C-MOF/ES-3 and C-MOF/EB-1 at c.a. 24°, while the peak of the carbon phase is below the observable level in the diffractograms of the samples C-MOF/EB-2 and C-MOF/EB-3, which contain the highest amount of noncarbonaceous phase among all composites (c.a. 68 wt.%, as found from TGA, see Section 3.5).

It can be concluded that XRPD patterns of the C-MOF/EB series, with the noncarbonaceous part dominating over the carbonaceous part, revealed that the noncarbonaceous (Zn-containing) part of all three samples is the wurtzite ZnO phase only. On the other hand, for the composites of the C-MOF/ES series, with the carbonaceous part dominating over the noncarbonaceous part, XRPD patterns confirmed the presence of wurtzite-type ZnS phase in all samples and wurtzite ZnO phase in the samples C-MOF/ES-2 and C-MOF/ES-3.

By applying Scherrer’s formula:

$$L=\frac{K\lambda }{FWHM\;\cos\theta }$$

where *L* is crystallite size (in nm), *K* is constant (≈0.9), *λ* is wavelength, *θ* is position of reflection on diffractogram (in radians), and *FWHM* is Full Width at Half Maximum for reflection (in radians), the crystallite size was determined for the samples whose XRD patterns were suitable enough. The calculated crystallite size for ZnO phase amounted to 40 nm, 30 nm, 34 nm, and 35 nm for the samples C-MOF/EB-1, C-MOF/EB-2, C-MOF/EB-3, and C-MOF/ES-3, respectively. We need to keep in mind that Scherrer’s formula almost always underestimates value for the crystallite size. The proposed route for the formation of ZnS during the carbonization is via a reaction of Zn^2+^ ions from MOF-5 with SO_4_^2−^ and HSO_4_^2−^ counter-ions from the PANI-ES part of the composite precursor for C-MOF/ES series, leading to ZnSO_4_, which was further reduced to ZnS by the released carbon species. Since SO_4_^2−^ and HSO_4_^2−^ ions are not present in the PANI-EB part of the precursors for the C-MOF/EB series, the appearance of the ZnS phase in the samples of that series is thus not expected, in line with experimental findings. The most probable reason not to observe characteristic ZnO peaks only in the diffractogram of the C-MOF/ES-1 sample is that the amount of this phase is below the detection limit of the XRPD technique. On the other hand, EDX elemental analysis of this sample suggested that the part of total Zn is bonded with S within ZnS, whereas the rest of Zn (the major part of total Zn) is bonded with O in ZnO phase (Table 2 and Table S1).

### 3.5. Thermal Analysis of C-(MOF-5/PANI) Samples—TGA and DTA

**Figure 5:**
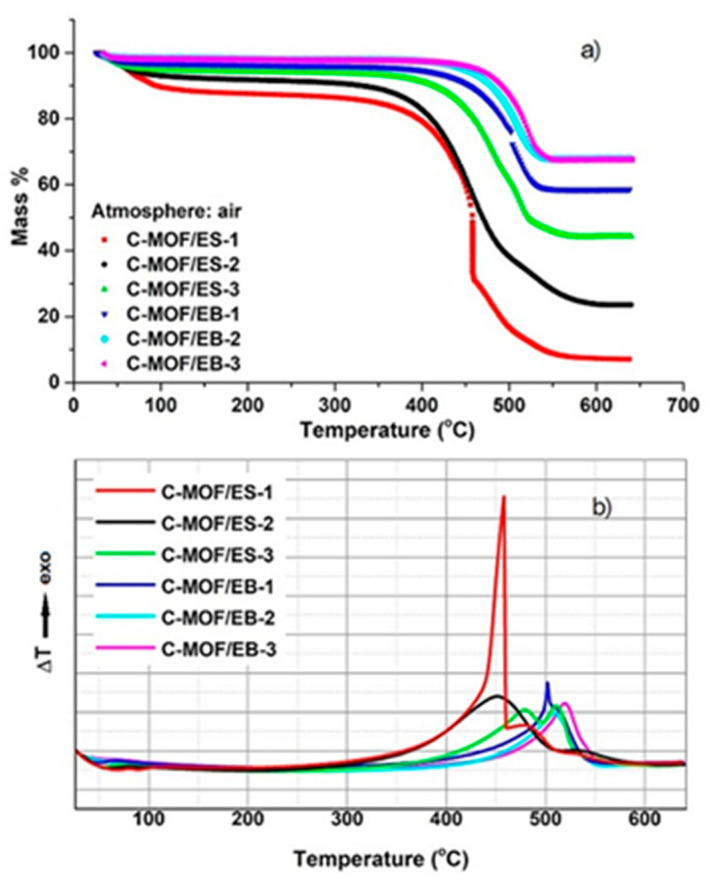
(**a**) TGA and (**b**) DTA curves of C-(MOF-5/PANI) samples, recorded in an air stream.

TGA curves obtained by heating samples in an air stream are shown in Figure 5a.

The first mass loss from 25 °C to c.a. 150 °C can be attributed mainly to the release of the surface adsorbed moisture. This mass loss is the largest for the sample C-MOF/ES-1, c.a. 10 wt.% at 100 °C, and decreased with decreasing the content of PANI in the precursors, i.e., with decreasing N content in C-(MOF-5/PANI) samples, implying that the presence of N improved the material’s hydrophilicity. This result is in agreement with previous results on carbonized PANI nanotubes, N-CNTs, which exhibit mass loss of 10–20 wt.% below 100 °C in air, in contrast to ordinary CNTs which showed a negligible mass loss in the same temperature region [23]. The next, much larger mass loss, which corresponds to the combustion of the carbonaceous part in all the samples and conversion of ZnS to ZnO in the samples of the C-MOF/ES series, occurs in the temperature interval 350–570 °C for the C-MOF/ES series and 450–550 °C for the C-MOF/EB series, and after that, the plateau is observed. Finally, when the maximum temperature of 640 °C is reached, the combustion of the carbonaceous part of the samples is completed, and a white inorganic residue remains, representing ZnO. Thus, based on TGA data, the mass ratio of the carbonaceous part vs. the Zn-containing part in C-(MOF-5/PANI) composites was determined using the formula:

$$\frac{residual\;mass\;at\;c.a.\;400\;{}^{\circ}C\;\left(\%\right)-residual\;mass\;at\;640\;{}^{\circ}C\;\left(\%\right)}{residual\;mass\;at\;640\;{}^{\circ}C\;\left(\%\right)\;}$$

and the percent content of the carbonaceous part, Zn-containing part (ZnO for C-MOF/EB, ZnO+ZnS for C-MOF/ES series), and water in the composites was calculated. These data are presented in Table 3.

It can be seen that the carbonaceous part is dominating (77.83–50.10 wt.%) over the ZnO+ZnS part (6.97–42.28 wt.%) in the samples of the C-MOF/ES series, while the ratio is reversed in the C-MOF/EB series, where the ZnO phase is dominating (58.50–67.61 wt.%) over the carbonaceous part (35.66–28.19 wt.%). In each series, the content of the carbonaceous part decreases with increasing the content of MOF-5 (decreasing the content of PANI) in the precursor. These results are consistent with the results of elemental microanalysis. Therefore, it can be concluded that the composition of carbonization products can be adjusted by tuning the composition of MOF-5/PANI composite precursors.

Corresponding DTA curves (Figure 5b) show one exothermic peak for C-MOF/EB series samples, located in the temperature range 500–520 °C, which is related to the combustion of the carbonaceous part of the samples. On the other hand, DTA curves of C-MOF/ES series samples showed three exothermic peaks in the case of C-MOF/ES-1 (at 460, 480 and 540 °C) and two exothermic peaks in the case of C-MOF/ES-2 (450, 540 °C) and C-MOF/ES-3 (480, 510 °C). More complex DTA curves for C-MOF/ES samples are explained by the presence of an additional ZnS phase and its conversion to ZnO via the reaction of ZnS with available atmospheric oxygen

2ZnS + 3O_2_ → 2ZnO + 2SO_2_

which occurs at c.a. 500 °C [54]. Thus, the peak observed at 510 and 540 °C could be attributed to this reaction. The peak located at 450–480 °C is attributed to the combustion of the carbonaceous phase in C-MOF/ES samples, supported by the fact that its intensity decreases with decreasing the content of the carbonaceous phase in the sample (from sample “1” to sample “3”).

### 3.6. Electrical Conductivity of C-(MOF-5/PANI) Composites and Carbonized Individual Components of Their Precursors

All C-(MOF-5/PANI) composites showed high electrical conductivity (σ) in the range of 0.08–0.24 S cm^−1^, which is 2 to 6 orders of magnitude higher than the conductivity of corresponding MOF-5/PANI precursors (Table 4). Even by the carbonization of nonconducting MOF-5/PANI composite precursors (σ~10^−7^ S cm^−1^, Table 4 [43]), electroconductive C-(MOF-5/PANI) materials were produced. Interestingly, within both composite series, the highest conductivity showed the samples with the highest content of Zn-containing phase(s), produced from the precursors with the highest content of MOF-5: C-MOF/ES-3 (σ = 0.21 S cm^−1^) and C-MOF/EB-3 (σ = 0.24 S cm^−1^), Table 4. A drastic increase in the electrical conductivity of material upon heating treatment is explained by the structural transformation of the PANI component and BDC linkers in the MOF-5 component of the composite precursors into the highly conducting N- and O-doped graphitic structures during heating in an argon atmosphere. The flat surfaces of MOF-5 cubic crystals within MOF-5/PANI precursors played an important role in obtaining flat carbonized structures with high electrical conductivity. The formation of carbonization products from PANI layers covering surfaces of MOF-5 particles was dictated by the shape of these particles. Thus, MOF-5 served as a self-degrading template. This interpretation is also consistent with the highest conductivity found for the samples produced from the precursors with the highest amount of MOF-5, as these precursors contain the highest amount of flat templating surfaces. In addition, the ZnO and ZnS phases are semiconductors that also contribute to the total electrical conductivity of final C-(MOF-5/PANI) composites.

The electrical conductivity of the carbonized individual components of MOF-5/PANI precursors, C-MOF, C-PANI-ES, and C-PANI-EB, was also measured for comparison, and it amounted to 0.92, 0.66, and 0.87 S cm^−1^, respectively (Table 4). The presence of ZnO and ZnS phases led to decreased conductivity of C-(MOF-5/PANI) composites compared with the conductivity of C-PANIs, but the order of magnitude was preserved (Table 4). The decrease in conductivity is expectable, owing to poor room temperature electron conductivity of bare ZnO (~10^−7^ S cm^−1^ [55]) and ZnS (~10^−6^ S cm^−1^ [56]) in their undoped forms. On the other hand, the lower conductivity of C-(MOF-5/PANI) composites compared with C-MOF (Table 4) could be explained by the lower degree of structural order in MOF-5/PANI composite precursors compared with that in MOF-5, as a result of which the order/alignment of formed graphitic layers/crystallites in C-MOF was better.

### 3.7. Specific Surface Area of C-(MOF-5/PANI) Composites and Carbonized Individual Components of Their Precursors—N_2_ Physisorption Measurements

The specific surface area (S_BET_) of C-(MOF-5/PANI) composites and carbonized individual precursor components (C-PANI-ES, C-PANI-EB, and C-MOF) was determined by N_2_ physisorption measurements (Table 4). The samples of the C-MOF/EB series showed higher S_BET_ values than the samples of the C-MOF/ES series. This is in accordance with higher S_BET_ values of MOF/EB composite precursors (c.a. 1600–2700 m^2^ g^−1^) compared with S_BET_ of MOF/ES precursors (220–860 m^2^ g^−1^) [43]. With increasing the content of MOF-5 in the precursor (going from sample “1“ to sample “3”), S_BET_ increases for the C-MOF/ES series, from 47 to 393 m^2^ g^−1^, and decreases for C-MOF/EB series, from 609 to 412 m^2^ g^−1^. Among all as-prepared composites, the sample C-MOF/EB-1 showed the highest S_BET_ (609 m^2^ g^−1^), which is even higher than S_BET_ of C-MOF (553 m^2^ g^−1^). If one tries to make a correlation with the morphology of the C-(MOF-5/PANI) samples (Section 3.3), it can be deduced that the samples with a larger amount of cuboid particles, i.e., C-MOF/ES-3, C-MOF/EB-1, C-MOF/EB-2, and C-MOF/EB-3 (synthesized from the precursors in which MOF-5 was dominating, >70 wt.%), exhibited high values of S_BET_, 393, 609, 470, and 412 m^2^ g^−1^, respectively. Moreover, these values are higher than the S_BET_ of carbonized individual PANI components, C-PANI-ES (351 m^2^ g^−1^) and C-PANI-EB (273 m^2^ g^−1^). On the other hand, the composites with prevalently granular morphology, C-MOF/ES-1 and C-MOF/ES-2, produced from the precursors with the lowest content of MOF-5 (c.a. 25 and 50 wt.%), showed relatively low S_BET_ values, 47 and 109 m^2^ g^−1^, respectively. These features can be explained by the large porosity of the MOF-5 part in the precursors, leading to a more developed pore structure (and consequently higher S_BET_) of the C-(MOF-5/PANI) samples which were produced by the carbonization of precursors with high content of MOF-5.

### 3.8. Raman Spectra of C-(MOF-5/PANI) Composites

**Figure 6:**
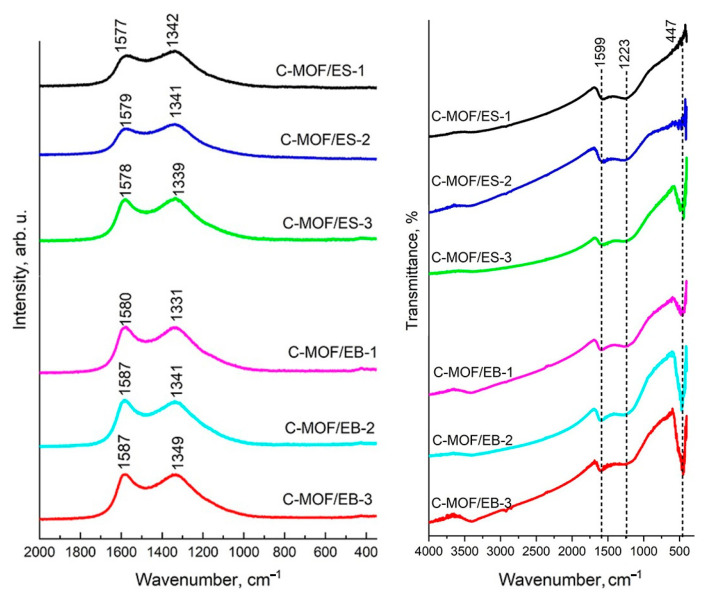
Raman spectra (**left**) and FTIR spectra (**right**) of C-(MOF-5/PANI) composites.

The Raman spectra of the as-prepared C-(MOF-5/PANI) composite samples (Figure 6, left) are simple, showing two broad bands in the 2000–400 cm^−1^ region, at 1577–1587 cm^−1^ (the graphitic G band) and 1331–1349 cm^−1^ (the disorder-induced D band), typical for disordered graphite and other disordered carbon materials [57,58]. These two bands and the absence of characteristic peaks of PANI [59] and MOF-5 [60] indicate that organic parts of MOF-5/PANI precursors were completely carbonized. The G band has been assigned to the stretching vibration of all pairs of sp^2^ atoms within the graphitic structure [57]. The D band has been attributed to the breathing vibrations of sp^2^ atoms in rings [57] and proposed to arise from graphene-layer carbon atoms adjacent to a lattice defect, such as the edge of the graphene layer or the heteroatom [58]. The Raman spectrum of perfectly ordered graphite (undisturbed, ideal graphitic lattice) in the 2000–400 cm^−1^ region shows only one sharp, strong band—the G band at around 1580 cm^−1^ [57,58]. The appearance of the additional D band has been associated with structural defects and the reduction in symmetry, which can be due to the incorporation of hetero-atoms into the graphitic pattern. The intensity ratio of the D and G bands (I_D_/I_G_) has been commonly used to evaluate the degree of disorder in carbon materials. The D band increases in intensity relative to the G band with increasing disorder degree [58]. In the Raman spectra of all C-(MOF-5/PANI) composite samples, the D band is significantly stronger than the G band. The intensity ratio I_D_/I_G_, determined using integrated peak areas upon the deconvolution of the spectra, was found to be in the range 3.42–3.67 (Table 4), indicating a significant level of disorder caused primarily by the incorporation of N and O in the carbon sp^2^ network. These I_D_/I_G_ values are similar to those found for N-doped carbon materials produced by the carbonization of PANI salts doped with different counterions (e.g., 3.48, 3.68, and 3.56 for carbonized PANIs doped with HSO_4_^−^/SO_4_^2−^, 3,5-dinitrosalicylate, and 5-sulfosalicylate anions, respectively [20]). For both the C-MOF/ES and C-MOF/EB series, the highest I_D_/I_G_ ratio was found for the samples with the lowest electrical conductivity (3.67 and 3.65 for C-MOF/ES-2 and C-MOF/EB-2, respectively). For the C-MOF/ES series, I_D_/I_G_ increases with decreasing electrical conductivity.

The Raman spectra of C-(MOF-5/PANI) samples do not exhibit the bands of ZnO and ZnS. The Raman spectrum of wurtzite ZnO shows a characteristic strong band at c.a. 440 cm^−1^ [61], while wurtzite ZnS shows weak Raman bands in the range 600–700 cm^−1^ [62]. The absence of these bands in the spectra of C-(MOF-5/PANI) composites can be explained by their overlapping with much stronger bands of the carbonaceous phase and by a relatively low surface concentration of ZnO and ZnS compared with the surface concentration of the carbonaceous part, supported by the fact that the depth resolution of Raman measurements was less than 0.7 μm and by the findings of elemental analyses regarding Zn content (Section 3.1 and Section 3.2).

### 3.9. FTIR Spectra of C-(MOF-5/PANI) Composites

FTIR spectra of all C-(MOF-5/PANI) composites (Figure 6, right) show two strong, broad bands, characteristic of disorder graphitic materials: the band at about 1599 cm^−1^, attributed to aromatic C=C stretching vibration (in sp^2^ configuration) and C=N stretching vibration (in N-doped carbons), and the band at about 1223 cm^−1^, attributed to mixed contributions of CC stretching (in disordered sp^3^ configuration), C-N, O-H, and C-O-C stretching vibrations [22,25,63]. It is known that pristine graphite, graphene, or carbon nanotubes do not show noteworthy IR signals, but only weak peaks due to adsorbed water may appear [64]. Incorporated heteroatoms (in our case, N and O) break the symmetry of the carbon network and cause the two mentioned broad bands to be active [22]. The characteristic bands of PANI [25,46] and MOF-5 [38] are absent in the spectra of C-(MOF-5/PANI)s, confirming the complete carbonization of organic parts in the precursors (PANI and BDC linkers).

In addition to the mentioned two bands of the carbonaceous phase, in the 2000–400 cm^−1^ region, the FTIR spectra of C-(MOF-5/PANI) composites show the characteristic sharp band of ZnO at c.a. 447 cm^−1^, which is assigned to Zn-O stretching vibration [48,49,63]. For both series, its intensity increases with the amount of MOF-5 in the precursor, i.e., with increasing the content of the noncarbonaceous phase in the C-(MOF-5/PANI) sample (determined by TGA, Table 3), as expected. The FTIR measurements cannot detect ZnS because its bands do not appear in the applied wavenumber range (a characteristic IR band of ZnS appears at about 300 cm^−1^ [63]. A broad absorption over the whole wavenumber range of 400–4000 cm^−1^ is observed in the FTIR spectra of all composites. This feature has also been observed in the FTIR spectra of other heteroatom-doped carbons [22,25] and attributed to the excitation of free (high-mobility) electrons, indicating the high electrical conductivity of synthesized materials. The broad band at c.a. 3400 cm^−1^ corresponds to O-H and N-H stretching vibrations [22,63].

### 3.10. Cyclic Voltammetry and Specific Capacitance of C-(MOF-5/PANI) Composites

**Figure 7:**
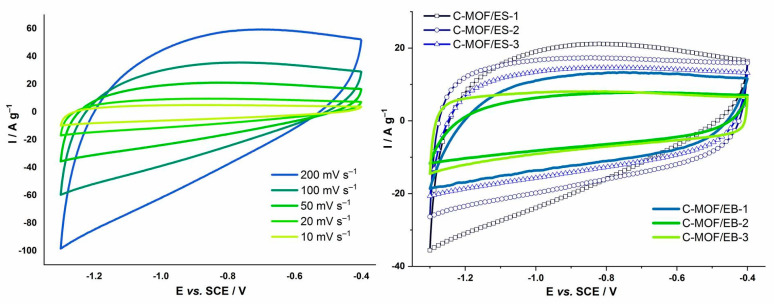
Cyclic voltammograms of the sample C-MOF/ES-1 measured at different potential sweep rates (v = 10, 20, 50, 100, and 200 mV s^−1^) (**left**) and comparison of CVs between C-(MOF-5/PANI) composite samples at v = 50 mV s^−1^ (**right**).

Cyclic voltammetry was used to evaluate the electrochemical behavior of C-(MOF-5/PANI) materials, particularly to assess charge storage ability and measure specific capacitance, C_spec_. Cyclic voltammograms (CVs) are measured in an alkaline (6 M KOH) electrolytic solution at various potential sweep rates (v) from 10 to 200 mV s^−1^, as shown for C-MOF/ES-1 composite in Figure 7 left. The values of gravimetric C_spec_ for all as-prepared C-(MOF-5/PANI) composites calculated from their CVs, at various v, are presented in Table 5. Recorded CVs are featureless, indicative of predominantly electrical double-layer capacitor (EDLC) behavior with no broad/sharp maxima hinting at pseudocapacitance and/or faradaic processes. Upon increasing the sweep rate, a slight positive slope is evidenced with a dip in the current at more negative potentials, pointing to the increase in the iR drop, probably stemming from the limited conductivity of the electrode material or constrained ion diffusion inside the pores, Figure 7 left.

The composites of the C-MOF/ES series showed higher C_spec_ (146.3–238.2 F g^−1^ at v = 10 mV s^−1^) than the composites of the C-MOF/EB series (91.2–136.2 F g^−1^ at v = 10 mV s^−1^), Table 5. The highest C_spec_ (238.2 F g^−1^ at v = 10 mV s^−1^) among all C-(MOF-5/PANI) composites exhibited the sample C-MOF/ES-1, prepared from the precursor with the lowest content of MOF-5. Within the C-MOF/ES series, for each v, C_spec_ decreased with increasing the content of MOF-5 in the precursor (from sample “1” to sample “3”). Similarly, for the C-MOF/EB series, the sample prepared from the precursor with the lowest content of MOF-5, C-MOF/EB-1, exhibited the highest C_spec_ (136.2 F g^−1^ at v = 10 mV s^−1^). However, no noticeable trend of C_spec_ was observed within that series.

A comparison of CVs between two series of composites at the same v of 50 mV s^−1^ is shown in Figure 7 (right). Interestingly, samples prepared from the precursors with the lowest amount of MOF-5 (samples “1”) in both series exhibited the largest capacitances with more tilted CVs, indicating some similarities which most probably come from the highest content of PANI in the structure before carbonization, and, consequently, the highest content of the carbonaceous part within the same series.

Noticeable differences in C_spec_ between the C-MOF/ES and C-MOF/EB series can be explained by the differences in structure and composition of MOF-5/PANI precursors used for their preparation and, consequently, by the differences in the relative content of carbonaceous vs. ZnO (or ZnO+ZnS) phases, the difference in the type of the noncarbonaceous phase (ZnO or ZnO+ZnS), and the differences in the elemental compositions (especially of N and C) between these two series. By comparison of the data presented in Table 3 and Table 5, it can be observed that the highest value of C_spec_ (238.2 F g^−1^ at 10 mV s^−1^) among all samples has the sample C-MOF/ES-1 with the highest content of carbonaceous phase, 77.83 wt.%, and the lowest content of inorganic phase (ZnO, ZnS), c.a. 7 wt.%. The next largest C_spec_ showed C-MOF/ES-2 (176.5 F g^−1^ at 10 mV s^−1^) with 65.67% of the carbonaceous phase. Moreover, one can see that the C_spec_ values within the C-MOF/ES series decreased gradually with decreasing the content of the carbonaceous phase. On the other hand, in composites of the C-MOF/EB series, the ZnO phase dominates over the carbonaceous phase (they have a noticeably lower fraction of the carbonaceous phase compared with samples of the C-MOF/ES series). This feature is well correlated with their noticeably lower C_spec_ compared with samples of the C-MOF/ES series. Very close values of C_spec_ for C-MOF/EB-2 and C-MOF/EB-3 align with their very close content of the carbonaceous phase (Table 3). By comparison of the data presented in Table 1, Table 2 and Table 3, it can be deduced that the values C_spec_ of C-(MOF-5/PANI) samples are also commensurate with their elemental contents of C and N. As N (originating from the PANI part of the precursor) is incorporated covalently into graphitic structures of the carbonaceous phase, this trend is consistent with the mentioned correlation between C_spec_ and the mass percent of the carbonaceous phase. The highest value of C_spec_ (238.2 F g^−1^) showed the sample C-MOF/ES-1, which has the highest bulk contents of N (9.95 wt.%) and C (65.6 wt.%), Table 1, and also the highest subsurface content of N (18.0 wt.%) and C (64.2 wt.%), Table 2, among all C-(MOF-5/PANI) samples. Considering the relationship between C_spec_ and S_BET_ for C-(MOF-5/PANI) composites (Table 4), interesting (and at first glance surprising) features were observed: (i) the sample C-MOF/ES-1 with the highest C_spec_ (238.2 F g^−1^) showed the lowest S_BET_ (47.3 m^2^ g^−1^) among all the samples, (ii) the composites of the C-MOF/ES series with lower S_BET_ (47.3–393 m^2^ g^−1^) exhibited higher C_spec_ than the composites of the C-MOF/EB series having higher S_BET_ (412–609 m^2^ g^−1^), (iii) in the C-MOF/ES series, C_spec_ decreased with increasing S_BET_, and (iv) in the C-MOF/EB series, the highest C_spec_ (136.2 F g^−1^ at v = 10 mV s^−1^) showed the sample with the highest S_BET_ (609 m^2^ g^−1^), but there was no tight correlation between C_spec_ and S_BET_ in the whole series.

It can be concluded that the most important role in achieving high C_spec_ values for the studied C-(MOF-5/PANI) composites was played by the content of the carbonaceous phase and the contents of C and N (N-containing functional groups) incorporated in that phase, while the impact of their total S_BET_ was much less important. Similar findings were reported in earlier works on N-containing carbons, where high capacitance was measured despite relatively low S_BET_; for example, for N-containing carbonized 1D-nanostructured PANIs doped with 5-sulfosalicylic, sulfuric, and 3,5-dinitrosalicylic acids, C_spec_ amounted to 391, 182, and 111 F g^−1^ at 10 mV s^−1^ in 6 M KOH solution, while their S_BET_ amounted to 317, 322, and 441 m^2^ g^−1^, respectively [27]. Similarly, Yang et al. measured C_spec_ in KOH for N-containing carbon nanotubes produced by the carbonization of PANI nanotubes to the amount of c.a. 110 and 163 F g^−1^ for carbonization temperatures of 600 and 700 °C, while their S_BET_ were c.a. 254 and 46 m^2^ g^−1^, respectively [65]. Such results have been explained by several effects beneficial for C_spec_: high content of incorporated N, which improves the wettability of material, the presence of N- and O-containing functional groups, which are involved in pseudofaradaic reactions and produce pseudocapacitance besides EDLC, as well as balanced meso- and microporosity.

We can also notice that for both series of C-(MOF-5/PANI) composites, there is no correlation between their C_spec_ and electrical conductivity (Table 4). To estimate the influence of the composite’s inherent electrical conductivity on the capacitance, the sample C-MOF/ES-1 with the highest C_spec_ was mixed with Vulcan XC72, a common commercial additive with high conductivity (~2.8 S cm^−1^ [66]) and very low capacitance (18 F g^−1^ at 5 mV s^−1^ [67]), which therefore cannot increase the total capacitance but significantly increases the conductivity of the drop-casted film. The measured electrochemical response for the sample with Vulcan XC72 additive was analogous to that of the pure sample, with C_spec_ being even somewhat lower, 173.5 F g^−1^ vs. 238.2 F g^−1^ at 10 mV s^−1^; the percentage reduction of C_spec_ is in good agreement with the percentage content of Vulcan XC72 (20 wt.%) in ink. The weak dependence of C_spec_ on conductivity points to the fact that the pore structure probably determines the CVs tilting through ion diffusion resistance upon increasing the sweep rate. Additionally, material loading on the GC electrode was checked for its impact on the measured capacitances. For used loadings of 0.25 mg cm^−2^_geom_ and 0.50 mg cm^−2^_geom_, we obtained essentially equivalent values of C_spec_, bearing in mind the standard error of the measurement (around 6% discrepancy).

If one compares the C_spec_ of C-(MOF-5/PANI) composites with the C_spec_ of carbonized individual components of precursors C-MOF, C-PANI/ES, and C-PANI/EB (Table 5), the main findings are as follows. All composites of the C-MOF/ES series exhibited significantly higher C_spec_ values compared with C_spec_ of C-MOF (72.1 F g^−1^ at v = 10 mV s^−1^), even though these composites have noticeably lower S_BET_ compared with C-MOF (Table 4). This finding reveals the importance of the PANI component in the composite precursors. All composites of the C-MOF/ES series also showed significantly higher C_spec_ values compared with carbonized individual PANI/ES components of their precursors, C-PANI/ES. Two samples of that series, C-MOF/ES-1 and C-MOF/ES-2, showed higher C_spec_, and the third, C-MOF/ES-3, showed comparable values of C_spec_ compared to that of C-PANI/EB. Regarding C-MOF/EB series, the composite C-MOF/EB-1 showed significantly higher C_spec_ than C-MOF and C-PANI/ES and slightly lower C_spec_ than the C-PANI/EB sample. The other two samples, C-MOF/EB-2 and C-MOF/EB-3, have similar C_spec_ values as C-MOF and C-PANI/ES and lower C_spec_ than C-PANI/EB, which could be explained by a much higher content of ZnO phase (c.a. 68 wt.%) compared with the content of carbonaceous phase (c.a. 29 and 28 wt.%) in these composites (Table 3). The C-(MOF-5/PANI) composites showed higher C_spec_ values than the related composites of the type carbon/ZnS (120.7 F g^−1^ at v = 5 mV s^−1^, 29.6 F g^−1^ at v = 100 mV s^−1^ [68] and carbon/ZnO (145 F g^−1^ at v = 2 mV s^−1^ [69].

### 3.11. Characterization of the Acid-Etched Samples

The selected C-(MOF-5/PANI) composites, i.e., those with the highest C_spec_ (C-MOF/ES-1 and C-MOF/ES-2) and the highest S_BET_ (C-MOF/EB-1), as well as the sample C-MOF, were subjected to the postcarbonization acid etching treatment with concentrated H_2_SO_4_ to reduce the content of Zn-containing phase and evaluate possible effects on the material’s properties. As a result, the content of Zn, measured by FAAS, was reduced from 7.76, 15.96, and 41.53 wt.% in starting samples C-MOF/ES-1, C-MOF/ES-2, and C-MOF/EB-1, respectively (Table 1), to 6.73, 8.26, and 2.97 wt.% in acid-etched samples C-MOF/ES-1-a, C-MOF/ES-2-a, and C-MOF/EB-1-a, respectively. It can be observed that the percentage of residual Zn upon the acid treatment increases with increasing the content of the carbonaceous phase present in the initial sample, indicating that the carbonaceous phase hinders the removal of ZnO and ZnS from the C-(MOF-5/PANI) composites. Thus, the vast majority of the Zn-containing phase was removed only from the composite C-MOF/EB-1, which contained the lowest content (c.a. 36 wt.%, Table 3) of the carbonaceous phase. By the same treatment, ZnO was almost completely removed from the C-MOF sample, as Zn content in C-MOF-a amounted to 0.15 wt.% (as determined by FAAS).

The data on electrical conductivities and S_BET_ of acid-treated samples C-MOF/ES-1-a, C-MOF/ES-2-a, C-MOF/EB-1-a, and C-MOF-a are shown in Table 4, whereas their C_spec_ are given in Table 5. It can be seen that the S_BET_ values of acid-treated samples C-MOF/ES-1-a (45.2 m^2^ g^−1^) and C-MOF/ES-2-a (106 m^2^ g^−1^) were slightly lower than the S_BET_ values of their parent samples C-MOF/ES-1 (47 m^2^ g^−1^) and C-MOF/ES-2 (109 m^2^ g^−1^). This finding is in accordance with a small decrease in Zn content upon acid treatment. On the other hand, the sample C-MOF/EB-1-a exhibited drastically (c.a. twice) higher S_BET_ = 1148 m^2^ g^−1^ compared with the counterpart as-prepared sample C-MOF/EB-1 (609 m^2^ g^−1^). The C_spec_ of C-MOF/EB-1-a is also significantly increased to 341.0 F g^−1^ at v = 10 mV s^−1^, compared with the C_spec_ of C-MOF/EB-1 (238.2 F g^−1^ at v = 10 mV s^−1^), which coincides with a mentioned large increase in S_BET_. The value of 341.0 F g^−1^ for C-MOF/EB-1-a represents the highest C_spec_ measured here for synthesized and characterized materials. This value overcomes or is comparable to the C_spec_ of related materials, such as CNTs/ZnO composite (323.9 F g^−1^) [70], the resin-derived N-doped porous carbon (341.0 F g^−1^ at 1 A g^−1^) with very high specific surface area (2248 m^2^ g^−1^) [71], or MOF-derived carbon/reduced graphene oxide composite (370.9 F g^−1^ at 1 A g^−1^) [72]. The samples C-MOF/ES-1-a and C-MOF/ES-2-a exhibited lower values of C_spec_ compared with the corresponding parent samples C-MOF/ES-1 and C-MOF/ES-2, which is in line with the mentioned small decrease in S_BET_. A positive correlation between C_spec_ and S_BET_ values is also observed for carbon materials prepared by the carbonization of bare MOF-5 (C-MOF) and subsequent acid etching (C-MOF-a): both values are significantly higher for C-MOF-a (224.8 F g^−1^ at v = 10 mV s^−1^ and 1994 m^2^ g^−1^) than the corresponding values for C-MOF (72.1 F g^−1^ at v = 10 mV s^−1^ and 553 m^2^ g^−1^). In order to provide additional insight into the charge storage mechanism of the studied materials, we analyzed the capacitive response of the selected samples, specifically C-MOF/ES-1 and C-MOF/EB-1 and their acid-etched counterparts (C-MOF/ES-1-a and C-MOF/EB-1-b). By plotting log*i*(*E*) vs. log*v*, a straight line is obtained with the slope *b,* which is in between two limiting cases: 0.5 (for faradaic processes) and 1 (for capacitive charging). Here, we find that for all four analyzed samples, *b* values are above 0.7, indicating a significant contribution of capacitive charging (Figure S2). Moreover, for acid-etched samples, *b* values were found to be higher than for as-synthesized samples, pointing to a higher capacitive contribution.

**Figure 8:**
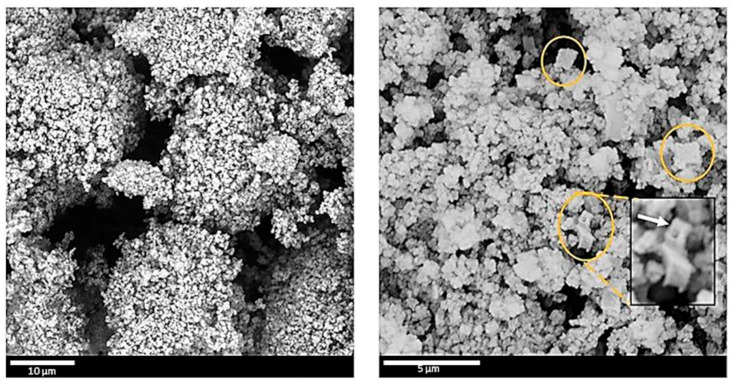
SEM images of the sample C-MOF/EB-1-a at magnifications 5000× (**left**) and 15,000× (**right**). Examples of cubical particles are circled; on the inset, a cubical particle with a cavity is marked by an arrow.

Due to acid etching, which caused a large reduction in the content of the ZnO phase, the electrical conductivity of C-MOF/EB-1-a noticeably increased to 0.42 S cm^−1^, compared with the electrical conductivity of the parent sample C-MOF/EB-1, 0.18 S cm^−1^ (Table 4). Similarly, samples C-MOF/ES-1-a and C-MOF/ES-2-a showed increased conductivities, 0.42 S cm^−1^ and 0.41 S cm^−1^, compared with conductivities of parent samples C-MOF/ES-1 (0.16 S cm^−1^) and C-MOF/ES-2 (0.10 S cm^−1^), respectively. On the other hand, C-MOF-a exhibited significantly lower conductivity (0.13 S cm^−1^) than the corresponding as-prepared C-MOF sample (0.92 S cm^−1^), which could be explained by the negative effect of ZnO removal on the stacking of graphitic layers in the remaining carbonaceous phase. The morphology of the sample C-MOF/EB-1-a, which showed the highest C_spec_, was studied by SEM, as shown in Figure 8. Compared with the morphology of the corresponding as-prepared C-MOF/EB-1 sample (Figure 2b), the morphology of C-MOF/EB-1-a is more granular and spongier, with visible cavities in many particles formed by the removal of ZnO using acid. An example of a cubical particle with a cavity is marked with an arrow in Figure 8 right.

## 4. Conclusions

In this work, we synthesized for the first time electroconducting composites of the type N,O-doped carbon/ZnO/ZnS and N,O-doped carbon/ZnO (named C-(MOF-5/PANI)) by the carbonization of the MOF-5/PANI composites. Two series of C-(MOF-5/PANI) composites, C-MOF/ES and C-MOF/EB, respectively, were derived from the MOF/ES and MOF/EB series of precursor composites synthesized with conducting PANI-ES form and nonconducting PANI-EB form, respectively, and using different MOF-5 to PANI mass ratios. Composites were characterized by elemental microanalysis, SEM, EDX, TGA/DTA, XRPD, AAS, FTIR and Raman spectroscopies, electrical conductivity, N_2_ physisorption, and cyclic voltammetry measurements. Individual components, MOF-5, PANI-ES, and PANI-EB, were also carbonized and characterized for comparison. XRPD and EDX showed that the carbonization of MOF/ES and MOF/EB precursors resulted in N,O-doped carbon/ZnO/ZnS and N,O-doped carbon/ZnO composites, respectively, in which ZnO and ZnS existed as pure hexagonal wurtzite-type crystalline phases, while the carbonaceous component was amorphous. TGA revealed the prevalence of the carbonaceous phase (77.83–50.10 wt.%) over the ZnO/ZnS phases in the C-MOF/ES series, and the prevalence of ZnO phase (58.50–67.61 wt.%) over the carbonaceous phase in C-MOF/EB series. In each series of produced composites, the content of the carbonaceous phase decreased with increasing MOF-5 content in the precursor. The bulk N content decreased in both series with decreasing PANI content in the precursor and amounted to 4.64–9.95 wt.% for the C-MOF/ES and 0.95–2.43 wt.% for the C-MOF/EB series. All C-(MOF-5/PANI) composites showed good electrical conductivity, in the range of 0.08–0.24 S cm^−1^, which was 2–6 orders of magnitude higher than that of MOF-5/PANI precursors due to the formation of conducting graphitic structures during the carbonization. Raman and FTIR spectra confirmed the complete carbonization of organic parts in MOF-5/PANI precursors. Raman spectra contain G and D bands, characteristic of disordered graphites, positioned at c.a 1580 cm^−1^ and 1340 cm^−1^, respectively, with the intensity ratio I_D_/I_G_ in the range 3.4–3.6, revealing a high extent of the disorder. For the C-MOF/ES and C-MOF/EB series samples, S_BET_ was in the range 47–392 m^2^ g^−1^ and 412–609 m^2^ g^−1^, respectively, and the highest S_BET_ (609 m^2^ g^−1^) showed C-MOF/EB-1 with the lowest content of ZnO phase (58.5 wt.%) within the C-MOF/EB series. Despite their lower S_BET_ values, N,O-doped carbon/ZnO/ZnS composites (C-MOF/ES series) showed noticeably higher C_spec_ (146.3–238.2 F g^−1^ at v = 10 mV s^−1^) than the N,O-doped carbon/ZnO (C-MOF/EB series) composites (91.2–136.2 F g^−1^ at v = 10 mV s^−1^), as measured by cyclic voltammetry in 6M KOH. This feature was attributed primarily to the higher content of covalently incorporated N in C-MOF/ES samples, which induces increased wettability and pseudocapacitance. Values of C_spec_ commensurate with the fraction of the carbonaceous phase and the content of N in the C-(MOF-5/PANI) composites, while the impact of S_BET_ was much less important. The highest C_spec_ (238.2 F g^−1^) among all as-synthesized composites exhibited the sample C-MOF/ES-1 with the lowest S_BET_ (47 m^2^ g^−1^) and the lowest content of ZnO/ZnS phase (c.a. 7 wt.%). Acid etching treatment of the C-MOF/EB-1 composite led to significantly increased values of C_spec_ and S_BET_, 341 F g^−1^ (at 10 mV s^−1^) and 1148 m^2^ g^−1^, respectively, accompanied with significantly reduced Zn content. That value of C_spec_ was the highest measured among the investigated materials, including carbonized individual components MOF-5, PANI-ES, and PANI-EB. Moreover, capacitive contribution to the current response was higher for acid-etched samples compared with their as-synthesized counterparts.

The advantage of the developed synthetic approach is that the composition and properties of produced C-(MOF-5/PANI) composites can be tuned by the composition of MOF-5/PANI precursors and the form of PANI in them. The PANI component served as a source of N heteroatoms, and its ES form with SO_4_^2−^ and HSO_4_^−^ counter-ions enabled the production of ZnS phase. The MOF-5 component served as a source of Zn^2+^ ions and as a self-transforming template, enabling the production of ZnO and ZnS phases and, when present in enough amount in the precursor, high S_BET_ of the final composite. Thus, PANI and MOF-5 acted synergistically to produce binary N,O-doped carbon/ZnO and ternary N,O-doped carbon/ZnO/ZnS composites with high electrical conductivity, specific surface area, and specific capacitance, which are desirable properties for supercapacitors and other energy storage and conversion applications.

## Figures and Tables

**Table 1:** Mass (m), the carbonization yield (calculated for 0.5 g of MOF-5/PANI precursor), and the bulk elemental composition of the as-prepared C-(MOF-5/PANI) samples determined by elemental microanalysis (C, H, and N), FAAS (Zn), and by difference (O).

C-(MOF-5/PANI) Sample	*m* (g)	Yield (%)	Content (wt.%)				
			C	N	H	Zn	O
C-MOF/ES-1	0.243	48.6	65.61	9.95	1.53	7.76	15.15
C-MOF/ES-2	0.222	44.4	53.97	8.30	1.42	15.96	20.35
C-MOF/ES-3	0.201	40.2	43.41	4.64	1.22	31.34	19.39
C-MOF/EB-1	0.216	43.2	35.82	2.43	1.05	41.53	19.17
C-MOF/EB-2	0.235	47.0	28.89	1.41	0.90	45.05	23.75
C-MOF/EB-3	0.241	48.2	28.84	0.95	0.77	44.90	24.54

Note: Sulphur was not detected—if present, its content was below the limit of detection (<0.4 wt.%).

**Table 2:** The elemental composition of C-(MOF-5/PANI) samples determined from EDX mapping.

C-(MOF-5/PANI) Sample	Content (wt.%)					
	C	Zn	N	O	Cl	S
C-MOF/ES-1	64.20	8.85	18.00	7.74	0.43	0.78
C-MOF/ES-2	59.32	13.48	16.40	9.55	0.57	0.68
C-MOF/ES-3	57.87	27.55	7.09	6.60	0.34	0.55
C-MOF/EB-1	74.77	5.72	7.40	11.68	/	0.43
C-MOF/EB-2	67.12	17.73	2.90	11.95	/	0.30
C-MOF/EB-3	57.05	33.03	2.03	7.77	/	0.12

**Table 3:** The content of the carbonaceous part, Zn-containing part (ZnO, ZnO+ZnS) part, and H_2_O, as well as the mass ratio of the carbonaceous part and Zn-containing part, (w_C/ZnO,ZnS_), in C-(MOF-5/PANI) composites, determined by TGA.

C-(MOF-5/PANI) Sample	Content (wt.%)			w_C/ZnO,ZnS_
	Carbonaceous Part	Zn-Containing Part (ZnO, ZnO+ZnS)	H_2_O	
C-MOF/ES-1	77.83	6.97	15.20	11.17
C-MOF/ES-2	65.67	23.52	10.81	2.79
C-MOF/ES-3	50.10	42.28	7.62	1.19
C-MOF/EB-1	35.66	58.50	5.84	0.61
C-MOF/EB-2	28.98	67.83	3.19	0.43
C-MOF/EB-3	28.19	67.61	4.20	0.42

**Table 4:** Electrical conductivity (σ), specific surface area (S_BET_), and the intensity ratio of Raman D and G bands (I_D_/I_G_) of C-(MOF-5/PANI) composites. Data for S_BET_ and σ for carbonized individual components of precursors and acid-etched samples, as well as σ of MOF-5/PANI composite precursors, are also presented.

**Precursors**		**Products of Carbonization**			
**MOF-5/PANI**	**σ ^a^** **(S cm^−1^)**	**C-(MOF-5/PANI)**	**σ** **(S cm^−1^)**	**S_BET_** **(m^2^ g^−1^)**	**I_D_/I_G_**
MOF/ES-1	1.0 × 10^−3^	C-MOF/ES-1	0.16	47	3.56
MOF/ES-2	1.4 × 10^−4^	C-MOF/ES-2	0.10	109	3.67
MOF/ES-3	7.9 × 10^−7^	C-MOF/ES-3	0.21	393	3.43
MOF/EB-1	4.3 × 10^−7^	C-MOF/EB-1	0.18	609	3.42
MOF/EB-2	3.9 × 10^−7^	C-MOF/EB-2	0.08	470	3.65
MOF/EB-3	4.0 × 10^−7^	C-MOF/EB-3	0.24	412	3.61
		**Carbonized Individual Components**			
		C-MOF	0.92	553	
		C-PANI-ES	0.66	351	
		C-PANI-EB	0.87	273	
		**Acid-Etched Samples**			
		C-MOF/ES-1-a	0.42	45.2	
		C-MOF/ES-2-a	0.41	106	
		C-MOF/EB-1-a	0.42	1148	
		C-MOF-a	0.13	1994	

^a^ data taken from ref. [41].

**Table 5:** The gravimetric specific capacitance (C_spec_) of C-(MOF-5/PANI) composites, carbonized individual components, and acid-etched samples, calculated from their CVs recorded at various potential sweep rates (10–200 mV s^−1^).

C-(MOF-5/PANI)	C_spec_ (F g^−1^)				
	10 mV s^−1^	20 mV s^−1^	50 mV s^−1^	100 mV s^−1^	200 mV s^−1^
C-MOF/ES-1	238.2	219.2	187.7	152.4	130.2
C-MOF/ES-2	176.5	169.4	158.8	143.2	129.0
C-MOF/ES-3	146.3	139.4	127.5	111.2	92.6
C-MOF/EB-1	136.2	127.6	110.5	90.3	68.3
C-MOF/EB-2	80.4	75.3	65.2	54.1	41.5
C-MOF/EB-3	91.2	83.3	74.7	63.4	54.1
**Carbonized Individual** **Components**	**10** **mV s^−1^**	**20** **mV s^−1^**	**50** **mV s^−1^**	**100** **mV s^−1^**	**200** **mV s^−1^**
C-MOF	72.1	69.2	67.4	61.1	60.5
C-PANI-ES	96.1	81.5	67.7	55.8	46.1
C-PANI-EB	144.7	139.0	126.4	107.7	89.7
**Acid-Etched Samples**	**10** **mV s^−1^**	**20** **mV s^−1^**	**50** **mV s^−1^**	**100** **mV s^−1^**	**200** **mV s^−1^**
C-MOF/ES-1-a	171.5	167.0	156.5	133.5	114.0
C-MOF/ES-2-a	163.9	160.0	157.1	133.2	114.1
C-MOF/EB-1-a	341.0	336.4	319.8	306.4	268.2
C-MOF-a	224.8	221.6	208.2	185.5	160.6

## Data Availability

Not applicable.

## Supplementary Materials

The following supporting information can be downloaded at: https://www.mdpi.com/article/10.3390/ma16031018/s1, Figure S1: EDX spectra of C-(MOF-5/PANI) samples of (A) C-MOF/ES series and (B) C-MOF/EB series; Table S1: Zn and O contents (total and in individual phases) of C-(MOF-5/PANI) samples calculated from EDX data; Table S2: Zn and O contents (total and in individual phases) of C-(MOF-5/PANI) samples, calculated from FAAS (total Zn content), elemental analysis (total O content), and EDX data (S content); Table S3: Phase assignation and indexing of reflections observed in XRPD patterns of C-(MOF-5/PANI) samples; Figure S2: The b-values for selected samples in a wide electrode potential range.
