# Supplementary material for: Carbonization of MOF-5/Polyaniline Composites to N,O-Doped Carbon/ZnO/ZnS and N,O-Doped Carbon/ZnO Composites with High Specific Capacitance, Specific Surface Area and Electrical Conductivity
Source: Materials (Basel). 2023 Jan 22;16(3):1018. doi: 10.3390/ma16031018 (PMC9919207; doi:10.3390/ma16031018)
Supplement: Supplementary file 1 [file materials-16-01018-s001.zip › materials-2139556-supplementary.pdf]

materials

Supplementary materials

Carbonization of MOF-5/Polyaniline Composites to N,O-Doped Carbon/ZnO/ZnS and N,O-Doped Carbon/ZnO Composites with High Specific Capacitance, Specific Surface Area and Electrical Conductivity

Marjetka Savić [1], Aleksandra Janošević Ležaić [2], Nemanja Gavrilov [3], Igor Pašti [3], Bojana Nedić Vasiljević [3], Jugoslav Krstić [4] and Gordana Ćirić-Marjanović [3,*]

1 Vinča Institute of Nuclear Science, University of Belgrade, National Institute of the Republic of Serbia, P.O. Box 522, 11001 Belgrade, Serbia
2 Faculty of Pharmacy, University of Belgrade, Vojvode Stepe 450, P.O. Box 146, 11221 Belgrade, Serbia
3 Faculty of Physical Chemistry, University of Belgrade, Studentski trg 12-16, 11158 Belgrade, Serbia
4 Department of Catalysis and Chemical Engineering, Institute of Chemistry, Technology and Metallurgy, University of Belgrade, Njegoševa 12, 11000 Belgrade, Serbia
* Correspondence: gordana@ffh.bg.ac.rs

Citation: Savić, M.; Janošević Ležaić, A.; Gavrilov, N.; Pašti, I.; Nedić Vasiljević, B.; Krstić, J.; Ćirić-Marjanović, G. Carbonization of MOF-5/Polyaniline Composites to N,O-Doped Carbon/ZnO/ZnS and N,O-Doped Carbon/ZnO Composites with High Specific Capacitance, Specific Surface Area and Electrical Conductivity. *Materials* **2023**, *16*, 1018. https://doi.org/10.3390/ma16031018

Academic Editor: Anastasios J. Tasiopoulos

Received: 19 December 2022
Revised: 12 January 2023
Accepted: 18 January 2023
Published: 9 February 2023

Copyright: © 2023 by the authors. Licensee MDPI, Basel, Switzerland. This article is an open access article distributed under the terms and conditions of the Creative Commons Attribution (CC BY) license (https://creativecommons.org/licenses/by/4.0/).

S1. EDX spectra of C-(MOF-5/PANI) samples

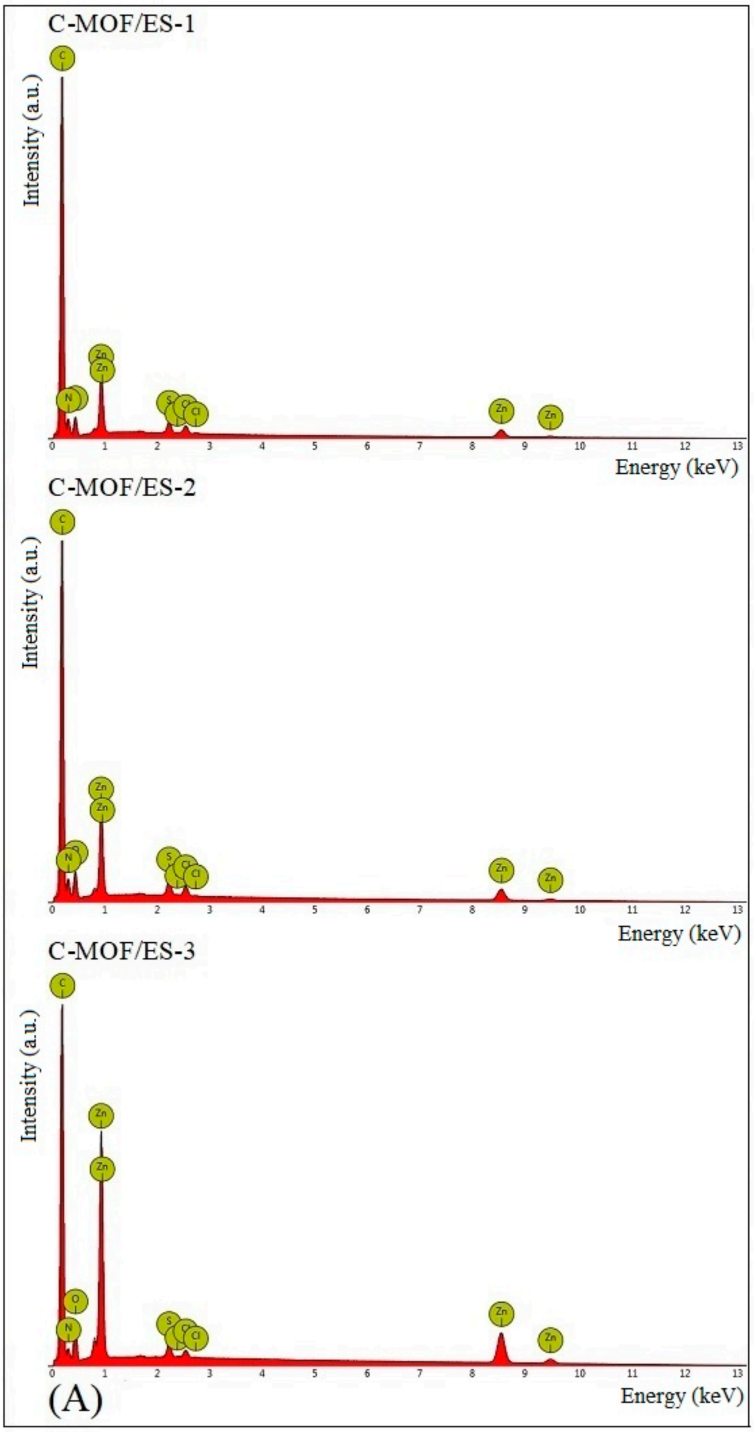

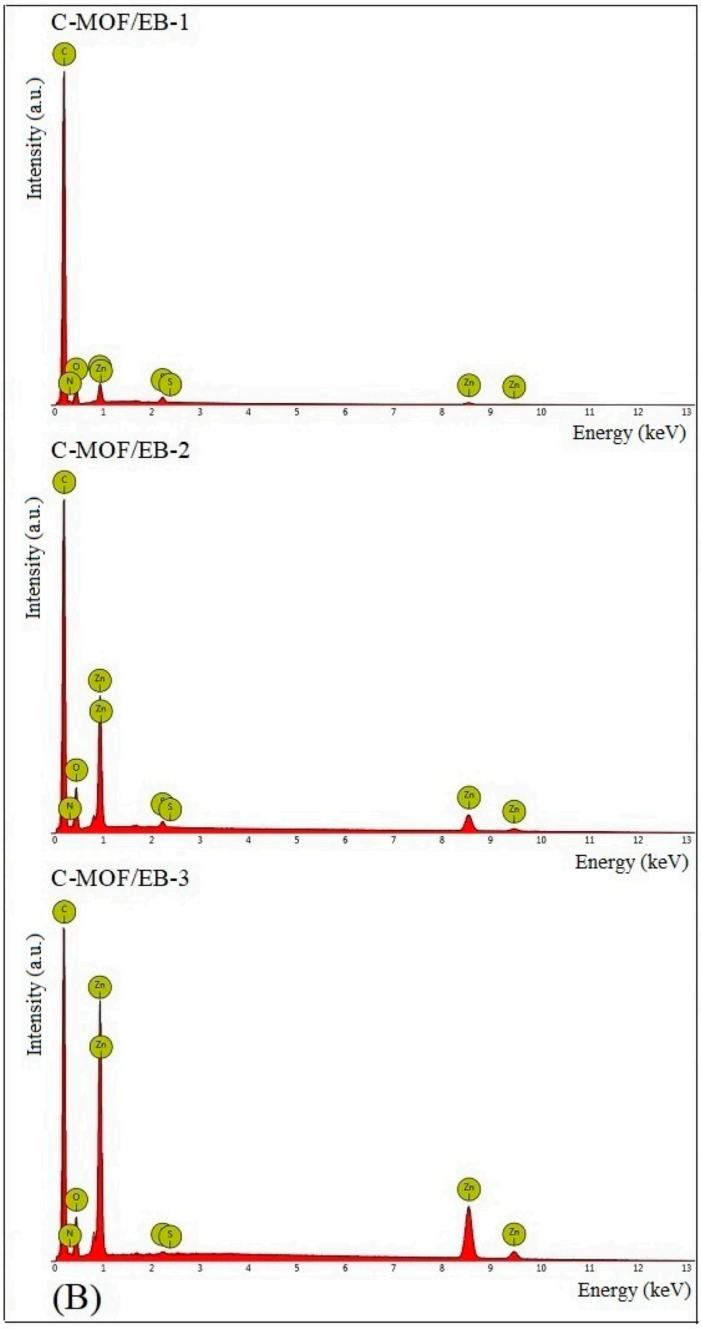

Figure S1. EDX spectra of C-(MOF-5/PANI) samples of (A) C-MOF/ES series and (B) C-MOF/EB series.

S2. Confirmation of O incorporation into the carbonaceous phase of C-(MOF-5/PANI) composites

Confirmation that the part of O is incorporated into the carbonaceous phase of C-(MOF-5/PANI) composites (besides the part of O which is present within ZnO) is obtained by using data on the elemental contents of C-(MOF-5/PANI) samples shown in **Table 1** and **Table 2,** and taking into account the results of XRPD analysis which confirmed the presence of ZnO and ZnS phases in C-MOF/ES and ZnO phase in C-MOF/EB series (section 3.4). For C-MOF/EB series, we have first calculated the stoichiometric quantity (wt.%) of O bonded to Zn within ZnO, and then made the difference between the total amount of O and the amount of O in ZnO, thus obtaining the amount of O incorporated in the carbonaceous phase. For C-MOF/ES series, the presence of ZnS phase must be

additionally taken into account, so we first calculated the amount of Zn present within ZnS based on the content of S acquired by EDX analysis. The remaining amount of Zn corresponds to ZnO phase and it enabled calculation of the amount of O in ZnO phase. The rest of calculation to obtain the amount of O incorporated in the carbonaceous phase is the same as that for C-MOF/EB series. The calculated amounts of O in ZnO and carbonaceous phase, as well as the amounts of Zn present in ZnO and ZnS phases are presented in **Table S1** (calculation made from EDX data) and **Table S2** (calculation based on FAAS, elemental analysis and EDX data).

Table S1. Zn and O contents (total and in individual phases) of C-(MOF-5/PANI) samples calculated from EDX data.

C-(MOF-5/PANI) sample	Content (wt.%)					
	Zn total	Zn in ZnS	Zn in ZnO	O total	O in ZnO	O in carbon phase
C-MOF/ES-1	8.85	1.59	7.26	7.74	1.78	5.96
C-MOF/ES-2	13.48	1.39	12.09	9.55	2.96	6.59
C-MOF/ES-3	27.55	1.12	26.43	6.60	6.47	0.13
C-MOF/EB-1	5.72	/	5.72	11.68	1.40	10.28
C-MOF/EB-2	17.73	/	17.73	11.95	4.34	7.61
C-MOF/EB-3	33.03	/	33.03	7.77	7.77	/

Table S2. Zn and O contents (total and in individual phases) of C-(MOF-5/PANI) samples, calculated from FAAS (total Zn content), elemental analysis (total O content), and EDX data (S content).

C-(MOF-5/PANI) sample	Content (wt.%)					
	Zn total	Zn in ZnS	Zn in ZnO	O total	O in ZnO	O in carbon phase
C-MOF/ES-1	7.76	1.59	6.17	15.15	1.51	13.64
C-MOF/ES-2	15.96	1.39	14.57	20.35	3.56	16.79
C-MOF/ES-3	31.34	1.12	30.22	19.39	7.39	12.00
C-MOF/EB-1	41.53	/	41.53	19.17	10.16	9.01
C-MOF/EB-2	45.05	/	45.05	23.75	11.02	12.73
C-MOF/EB-3	44.90	/	44.90	24.54	10.98	13.56

S3. Phase assignation and indexing of reflections observed in XRPD patterns of C-(MOF-5/PANI) samples

Table S3. Phase assignation and indexing of reflections observed in XRPD patterns of C-(MOF-5/PANI) samples.[a.]

C-MOF/ES						C-MOF/EB					
C-MOF/ES-1		C-MOF/ES-2		C-MOF/ES-3		C-MOF/EB-1		C-MOF/EB-2		C-MOF/EB-3	
2Θ/°		2Θ/°		2Θ/°		2Θ/°		2Θ/°		2Θ/°	
24.4	Carbon (amorphous)	25.1	Carbon (amorphous)	24.2	Carbon (amorphous)	24.0	Carbon (amorphous)				
27.0	ZnS(100)	27.4	ZnS(100)	27.0	ZnS (100)						
28.7	ZnS(002)	28.9	ZnS(002)	28.6	ZnS(002)						

30.3	ZnS(101)	30.6	ZnS(101)	30.6	ZnS(101)						
		31.8	ZnO (100)	31.8	ZnO (100)	31.6	ZnO (100)	32.0	ZnO (100)	32.0	ZnO (100)
		34.2	ZnO (002)	34.5	ZnO (002)	34.4	ZnO (002)	34.7	ZnO (002)	34.6	ZnO (002)
		36.2	ZnO (101)	36.3	ZnO (101)	36.2	ZnO (101)	36.5	ZnO (101)	36.5	ZnO (101)
47.8	ZnS(110)	47.6	ZnO (102), ZnS(110)	47.6	ZnO (102), ZnS(110)	47.4	ZnO (102)	47.8	ZnO (102)	47.8	ZnO (102)
56.6	ZnS(112)	56.6	ZnO (110), ZnS(112)	56.6	ZnO (110), ZnS(112)	56.5	ZnO (110)	56.8	ZnO (110)	56.8	ZnO (110)
		62.8	ZnO (103)	62.9	ZnO (103)	62.7	ZnO (103)	63.1	ZnO (103)	63.0	ZnO (103)
				66.4	ZnO (200) ZnS(104)	66.3	ZnO (200)	66.5	ZnO (200)	66.5	ZnO (200)
				68.0	ZnO (112)	67.8	ZnO (112)	68.1	ZnO (112)	68.1	ZnO (112)
				69.2	ZnO (201)	68.9	ZnO (201)	69.3	ZnO (201)	69.2	ZnO (201)
				72.6	ZnO (004) ZnS (203)	72.5	ZnO (004)	72.8	ZnO (004)	72.7	ZnO (004)
				77.0	ZnO (202) ZnS (211)	76.8	ZnO (202)	77.1	ZnO (202)	77.1	ZnO (202)
				81.5	ZnO (104)	81.3	ZnO (104)	81.6	ZnO (104)	81.5	ZnO (104)

[a]Reflections originating from ZnS phase are assigned using ref. [51] and reflections from ZnO phase are assigned using ref. [49].

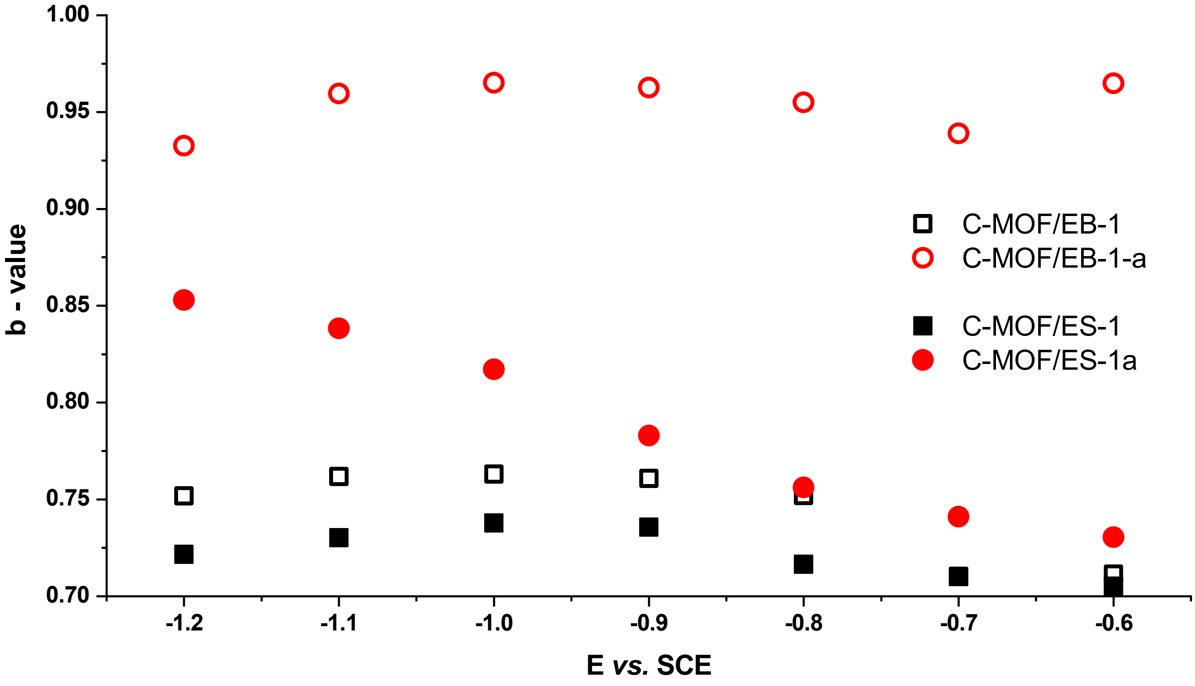

Figure S2. The *b*-values for selected samples in a wide electrode potential range.
